# An early Devonian flora from the Baviaanskloof Formation (Table Mountain Group) of South Africa

**DOI:** 10.1038/s41598-021-90180-z

**Published:** 2021-06-04

**Authors:** Robert W. Gess, Cyrille Prestianni

**Affiliations:** 1 Geology Department and Albany Museum, Rhodes University, Makhanda (Grahamstown), South Africa; 2 Evolution and Diversity Dynamics Laboratory (EDDy Lab), Geology Department, Liège University, Liège, Belgium; 3 OD Terres et Histoire de la Vie, Royal Belgian Institute of Natural Sciences, Brussels, Belgium

**Keywords:** Plant evolution, Biodiversity, Palaeoecology

## Abstract

Newly discovered early plant bearing lenses from the Baviaanskloof Formation at Impofu Dam in the Eastern Cape Province of South Africa provide evidence for one of the most diverse Late Silurian to Early Devonian assemblages known to date. This work represents the first account of this flora. Fifteen taxa are presented, including eleven diagnosed to existing genera, of which eight may be reasonably diagnosed to existing species including several species of the genus *Cooksonia*. Three new taxa, *Krommia parvapila*, *Elandia itshoba* and *Mtshaelo kougaensis* are described. This flora is furthermore remarkable for the large number of complete or sub-complete specimens allowing good understanding of earliest plant architecture. The assemblage bears the greatest resemblance to Early Lochkovian assemblages from the Parana Basin of Brazil and the Anglo Welsh basin. Biostratigraphic constraints on the dating of the Baviaanskloof Formation are provided by this flora, which represents the oldest known from Africa.

## Introduction

The late Silurian to earliest Devonian comprised a pivotal time in the evolution of early terrestrial ecosystems characterized by the progressive decline of cryptophytes (*sensu*^1^) and the rise of several lineages of trilete spore producing plants. Palaeontological evidence from this time is however very scarce and is as yet largely restricted to palaeotropical environments**.**

The only previously published high latitude floras from this time interval are those from the Paraná Basin of Brasil and Argentina ^2–6^, with a single plant species having been reported from southern Africa. This latter taxon, *Dutoitia pulchra*, was originally reported as being of Silurian age and as being from Bokkeveld Group strata exposed in the Blaaukrantz River valley in South Africa. Re-examination of the type locality by Robert Gess (RG) indicates that the plant was collected from a black shale unit within the Kareedouw Member of the Baviaanskloof Formation of the Table Mountain Group. Ongoing research (see below) suggests a probable Lochkovian age for this deposit.

**Figure 1:**
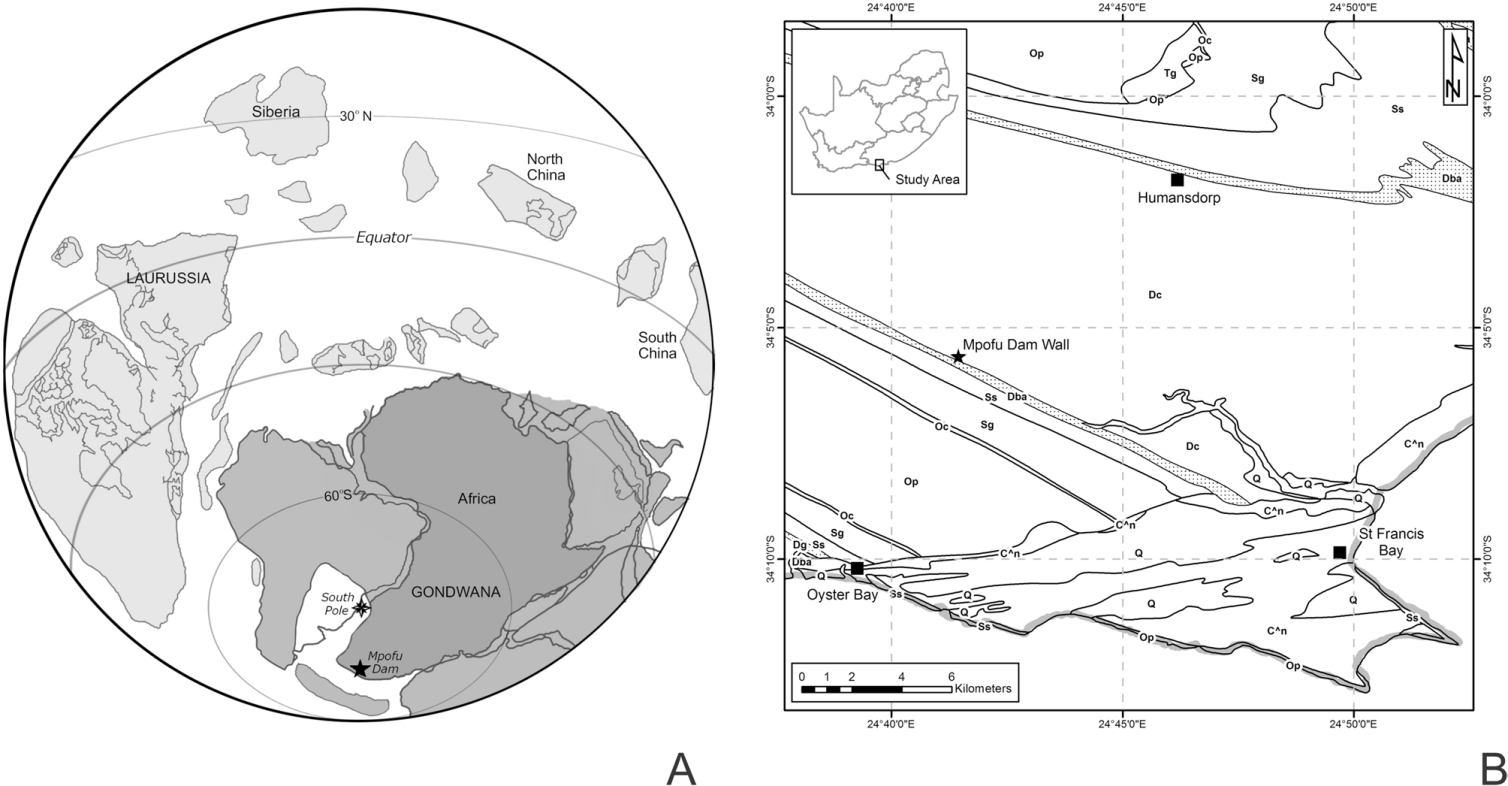
(**A**) Palaeogeographic situation of the Mpofu Dam locality. Map modified from Torsvik and Cocks^17^ (**B**) Map of the Humansdorp region showing the regional geology and the position of the Impofu Dam outcrops.

Herein we provide a detailed study of a diverse flora collected by RG from a formerly unknown locality within the same geological unit (the Kareedouw Member of the Baviaanskloof Formation) but located a hundred kilometres further to the east (Fig. 1). Comparison of the new flora with coeval assemblages from the Paraná (Brazil) and Anglo-Welsh Basins (United Kingdom) sheds new light on the formerly uncertain dating of the Baviaanskloof Formation, and by extension the age of the *Dutoitia* type material, as well as the transition from the Table Mountain Group to the overlying Bokkeveld Group.

## Geological setting

### The Baviaanskloof Formation

**Figure 2:**
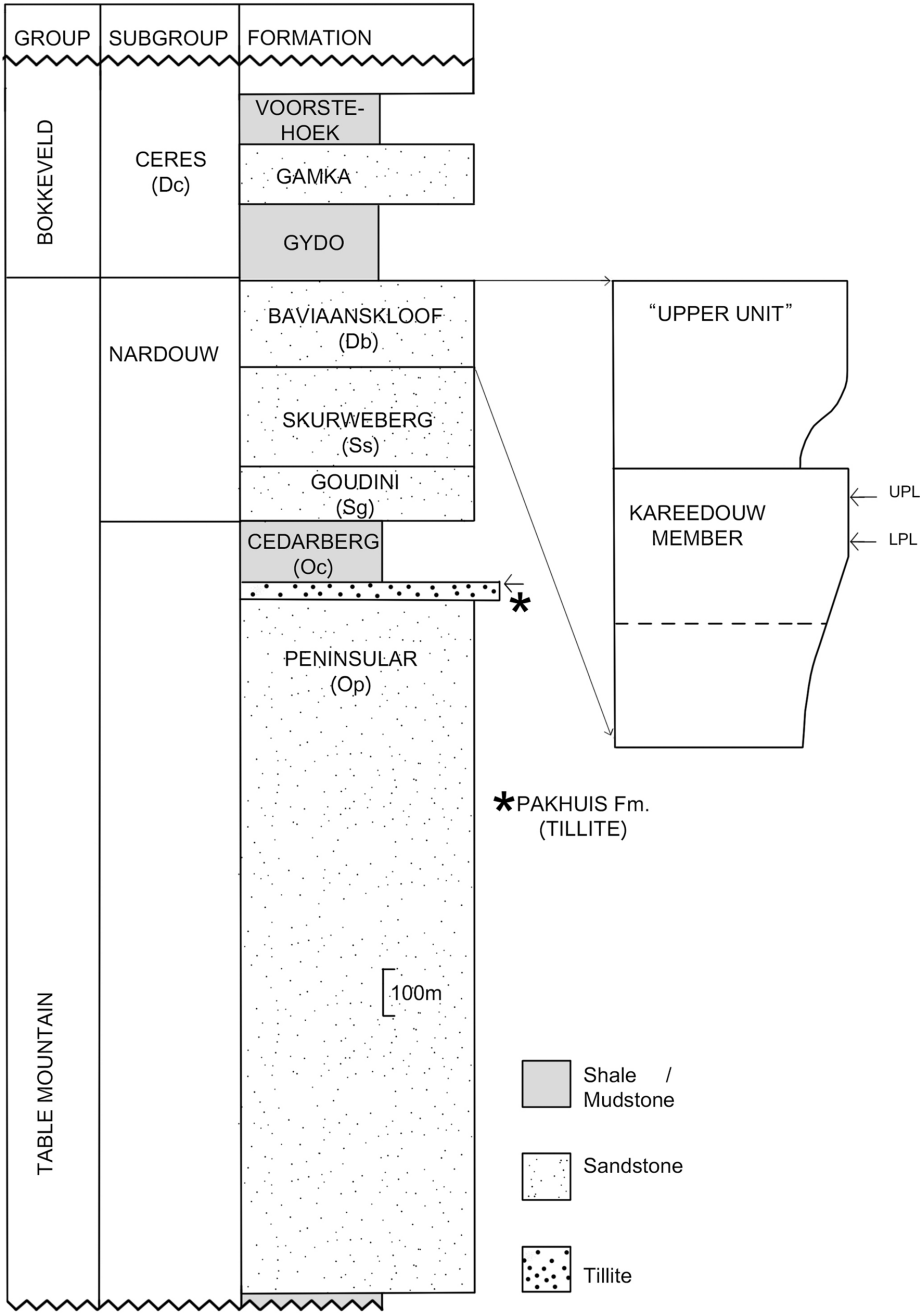
Stratigraphic column of the Impofu Dam locality.

The Baviaanskloof Formation (Table Mountain Group, Cape Supergroup), from which the material was recovered, is the uppermost formation of the Table Mountain Group and lateral equivalent to the more westerly occurring Rietvlei Formation. Originally termed the ‘passage beds’^70^ it comprises a transition between the clean quartzite dominated (Ordovician to Silurian) Table Mountain Group and the conformably overlying mudstone dominated (early to mid-Devonian) Bokkeveld Group (Fig. 2). The Baviaanskloof Formation coincides with deepening of the Agulhas basin due to both tectonic subsidence and global sea level rise. This resulted in a shift of deposition from predominantly fluvial and shallow shelf environments (during deposition of the Table Mountain Group) to fully marine settings (during deposition of the Bokkeveld Group). Prior to designation of the Baviaanskloof Formation^30,58^ these strata were often considered to form the basal part of the Bokkeveld Group^16,70^.

The Baviaanskloof Formation overlies the fluvially deposited Skuweberg Formation (Table Mountain Group) and is overlain by the marine mudstones of the Gydo Formation (Bokkeveld Group) (Fig. 2). The Baviaanskloof Formation predominantly comprises stacked, well-bedded, ‘dirty’, fine grained felspathic sandstones with interbedded mudstones. Though locally very variable it is generally considered to be divisible into unnamed ‘basal’ and ‘upper’ units separated by a cleaner quartzitic unit, the Kareedouw Member, which is of variable thickness^58^.

The upper unit contains marine invertebrates comprising homalonotid trilobites, a small range of articulate and inarticulate brachiopods, nuculid and other bivalves, plectonotid “gasteropods” and bryozoans^15,22,24,30,58,70^.

### Age of the Baviaanskloof Formation

As a result of a complete lack of stratigraphically useful microfossils or igneous horizons within the Table Mountain and Bokkeveld Groups, precise dating of their constituent formations has proved challenging. As yet no consensus has been reached regarding the precise age of the Baviaanskloof Formation, and by extension the onset of the rapid basinal deepening that ushered in deposition of the Bokkeveld Group—though Cooper^19^ correlated initiation of the Bokkeveld Group with the global Emsian transgression.

Biostratigraphically useful brachiopod remains are restricted to the ‘upper unit’ (Fig. 2), however the Baviaanskloof Formation represents an apparently conformable sequence deposited over some time with its lower two members having not, previously, yielded biostratigraphy clues. To some extent there has therefore been a tendency to derive ages for the whole formation from clues restricted to its uppermost member. Greater clarity has however been achieved during this study (see discussion section).

Historically, based on unspecified brachiopods from near the top of the Baviaanskloof Formation Theron^26^ considered it to be Early Devonian in age and suggested an Emsian age for the top of the Table Mountain Group^26^. Tankard et al.^71^ however considered it most likely that the Baviaanskloof Formation straddles the Silurian-Devonian boundary and ascribed an Emsian age to the overlying Gydo Formation (basal Bokkeveld Group) on the basis of abundant Malvinokaffric invertebrate fauna collected therein^71^.

An early Devonian age for the ‘upper unit’ of the Baviaanskloof Formation was supported by the presence of the mutationellid brachiopod, *Pleurothyrella* (Boucot, personal communication, 1981, quoted in Tankard et al.^71^). Presence of this genus in the ‘upper unit’ is indicative of a Pragian/Emsian age for these strata^13,20^. In his designation of the unit Hill^58^ endorsed the view of Tankard et al.^71^ that the Baviaanskloof Formation straddled the Silurian- Devonian boundary.

In reviews of the Cape Supergroup subsequent authors have presented a variety of opinions. Johnson^14^ and Theron^25^, for example, have illustrated the boundary between the Table Mountain Group and the Bokkeveld Group as congruent with the Silurian/Devonian boundary implying a latest Silurian age for the entire Baviaanskloof Formation. Thamm and Johnson^21^ diagrammatically represent the Silurian-Devonian boundary at the base of the Baviaanskloof Formation and suggest a Lochkovian to Emsian age for the Rietvlei Formation (the lateral equivalent to the Baviaanskloof Formation). Alternately Tankard et al.^71^ consider the Rietvlei Formation to be Pragian to Emsian in age.

For the first time we present diverse palaeontological material from the Kareedouw Member, which suggests a Lochkovian age for strata underlying the ‘upper unit’. Undescribed invertebrate remains from the ‘upper unit’ overlying the new plant fossil localities at Impofu Dam are consistent with the Pragian/Emsian age previously proposed for the ‘upper unit’^13,20^. We therefore believe the Baviaanskloof Formation to be Lochkovian to Pragian (or earliest Emsian) in age.

### The Impofu Dam locality

A previously unresearched section of the Baviaanskloof Formation in and below the spillway of the Impofu Dam in the Humansdorp district exposes a fairly complete section of the Baviaanskloof Formation, though it is tectonically somewhat distorted, making precise logging challenging and possibly leading to a degree of thickening. An estimated 265 m of stratigraphic thickness was measured. The stratigraphically lower part of the section initially comprises thick beds of impure greywacke corresponding to the ‘lower unit’, which grade upward into purer, cleaner, more resistant strata corresponding to the Kareedouw Member, the top of which is abruptly reached at about 60% of the stratigraphic thickness. Within this lower 60% of outcrop greywackes are interbedded with thin silty to fine grained black anaerobic micaceous mudstone lenses, (frequently degraded to a white colour), which extend from within the less pure greywackes up into the cleaner sandstones of the Kareedouw Member. This part of the section contains no marine invertebrate fossils; however, plant fragments are encountered in the anaerobic silty mudstones. Two such horizons have been found to be rich in plant fossils which sometimes form vegetation mats. Many examples are preserved in a near complete state, though the majority are fragmentary. The upper of these lenses (UPL) occurs 15 m below the top of the Kareedouw Member and the lower (LPL) occurs 24 m below that. They were found to contain similar floral elements though small differences in floral diversity are observed that may result from depositional factors, such as transport distance from their original habitat and differences in current strength. Plant fossils from the LPL are often more complete and finely preserved, particularly towards the top of the lens (which fines upwards). This suggests a lower energy, possibly more proximal setting, than that of the UPL. This lower portion of the section (below the top of the Kareedouw Member) is interpreted as representative of marginal marine depositional settings within which the anaerobic black lenses represent estuarine or back-barrier lagoonal deposits.

By contrast the overlying deposits of the ‘upper unit’ contain frequent stringers of brachiopods and interbeds of brownish, bioturbated mudstones, which are generally rich in shallow marine invertebrate remains and trace fossils. These are interpreted as representing shoreface and foreshore deposits with occasional shifts towards preservation of below wave base marine muds, which are silty and brown in colour.

## Methods

Specimens are preserved within a black metasediment derived, by low grade (lower greenschist facies) metamorphism, from a carbonaceous mudstone. Organic plant material is compressed and has all been replaced by silvery white phyllosilicate minerals. Specimens were prepared using sharp triangular needles and studied under a Zeiss Stemi 508 microscope. Photographs were taken dry with a Nikon D7500 camera utilising natural or normal electric light.

## Megafossil description

The fossils described in the following section have been collected from two different layers presenting slightly different assemblages but with several overlapping taxa. The descriptions are presented here in a taxonomic order. However, plates have been kept separate for the two localities. Plates 1–3 present the assemblage of the UPL while plates 4–9 present the plants that were collected from the LPL.

### Incertae sedis Bryophyta

*Sporogonites*^37^

*Sporogonites* sp. A

**Figure 3:**
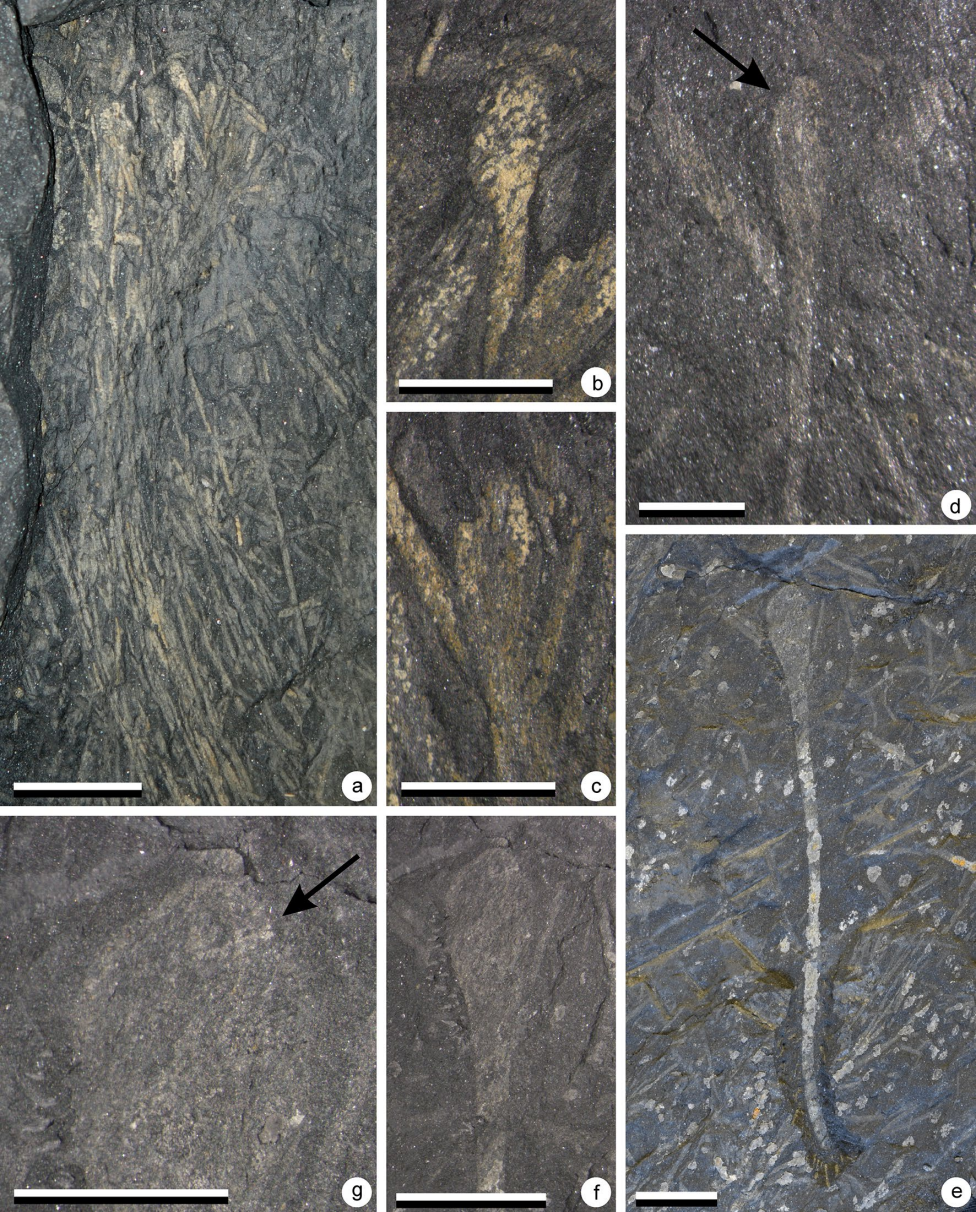
(**a**–**d**) *Sporogonites* sp. A, (**e**–**g**) *Sporogonites* sp*.* B., (**a**) Specimen AM 7944. Scale = 2 cm. Gross view of specimen. Several parallel slender non branching axes terminated by elongate sporangia. (**b**) Specimen AM 7944. Scale = 5 mm. Detail of the top part of the plant showing the shape of one sporangium. (**c**) Specimen AM 7944. Scale = 5 mm. Detail of the top part of the plant showing the shape of one sporangium. (**d**) Specimen AM 7944. Scale = 1 cm. Detail of an isolated specimen. Shape of one sporangium is visible. (**e**) Specimen AM 7953. Scales = 1 cm. Gross view of specimen showing the non-branching slender axis bearing terminally an elongate sporangium. (**f**) Specimen AM 7953. Scales = 1 cm. Detail of the sporangium. (**g**) Specimen AM 7953. Scales = 5 mm. Detail of the distal part of the sporangium characterized by a small notch (at arrow).

Fig. 3a–d; Fig. 7a–b

#### Material

This plant is very abundant in some layers where the elongated stems cover the whole bedding plane. Individual stems are in most cases difficult to identify.

#### Description

The plant consists of bunches of elongated non-divided axes, each ending in one sporangium when complete (Figs. 3a–d, 7a–b). In places, dichotomies seem to occur, but they result from the superimposition of axes. Axes are arranged in parallel. They are 10–11.5 cm long and 0.9–1.3 mm wide. Width is constant along their entire length.

The distal part of the stalk is marked by a progressive but clear flaring which identifies the position of the proximal part of the sporangium (Figs. 3b–d, 7b). Sporangia are 7–7.5 mm long and 3–3.5 mm wide.

Sporangia are elongated in shape and were probably ovoid to ellipsoid before compression. Their distal part is rounded in outline. The surface of the sporangia is unclear due to the coarse nature of the preservation. In some specimens, a small notch is observed on either side, approaching the tip of the sporangia, defining a small hemispheric structure (see arrows on Figs. 3d, 7b).

#### Identity and comparisons

The occurrence of elongated sporangia borne singly at the top of smooth unbranched axes unambiguously points to the genus *Sporogonites* Halle^27,37^. This genus is comprised of 4 (or 5) species: *S. exuberans* Halle^37^, *S. chapmanii* Lang and Cookson^33^, *S. excellens* Frenguelli^29^ and *S. yunnanense* Hsü^10^. An additional species was described by Gonez^31^ but was not validly published. Gonez^31^ named it *Sporogonites punctatus* in his unpublished PhD manuscript. However, according to the International Code of Nomenclature for algae, fungi and plants art. 30.9, this publication is not effective and hence not valid (op. cit., art. 32.1).

All species chiefly differ in the shape and size of their sporangia. *Sporogonites yunnanense* presents notably small sporangia ranging from 3.2–4.5 mm in length and 1.4–1.8 mm in width. By contrast, *S. excellens* is characterized by generally big sporangia up to 5 mm in width and 7 mm in length that are borne on up to 5 mm wide stalks. Our specimens do not compare favourably to either of these two species. The occurrence of a rounded apex to the sporangia in our specimens exclude them from *S. chapmanii* which is characterized by pointed sporangia. The above described South African specimens conform in shape and size range of the sporangia to *S. exuberans*. However, similar sporangia were previously reported as *Sporogonites* sp. A by Gerrienne et al.^2^ and as a *Sporogonites punctatus* in Gonez^31^ from the Paraná basin (Brazil). *Sporogonites exuberans* and *Sporogonites “punctatus*” differ mainly by the occurrence in the latter of a minute conical ornamentation on the upper half of the sporangia. The Brazilian material is comparable in size and shape to the Impofu Dam material, however the nature of the preservation of the latter precludes determination of the presence or absence of the diagnostic sporangial ornamentation. In order to avoid misleading paleogeographic interpretations we therefore prefer to leave the taxonomy of this plant open.

#### Age and distribution

The genus *Sporogonites* is a common component of the earliest floras. It is most frequently reported from Emsian aged deposits in which it constitutes a common and widespread taxon (for full list see^31^). Its oldest reported occurrences are from assemblages from the Late Silurian of Vietnam^31^. In the Lochkovian, it has thus far only been found in the Brazilian Ponta Grossa Formation^2,31^. It is noteworthy to mention that sporangial ornamentation aside, our material is comparable to this sole Lochkovian occurrence.

### Sporogonites

sp. B

Fig. 3e–g

#### Material

Only one specimen of this plant has been collected as a relatively well-preserved isolated stem.

#### Description

This plant consists of a long unbranched stem distally bearing a large elongated sporangium (Fig. 3e). The stem is straight and measures 66.0 mm long and 1.9–2.1 mm wide. The distal end of the stem is marked by a progressive widening corresponding to the beginning of the sporangium (Fig. 3f). From the point of widening to the tip, the sporangium measures 14.3 mm long and 6.1 mm wide. The sporangium reaches its maximum width after 9 mm (2/3 of total length). The sporangium is terminated by a hemispherical structure marked by a clear depression of the lateral outlines and demarcated by a line of denser mineralisation (see arrow on Fig. 3g). This structure is 3.4 mm wide and 2.1 mm high.

#### Identity and comparison

As for *Sporogonites* sp. A, the occurrence of an elongated sporangium borne singly on a smooth non branched axis suggests the genus *Sporogonites* Halle^37^. Lack of bifurcation cannot be unambiguously established as a result of the lack of preservation of the base. Nonetheless the size of the plant mitigates against other explanations. Moreover, the general shape of the sporangium conforms to the genus *Sporogonites*, being very similar to both *Sporogonites exuberans* and *Sporogonites “punctatus*”. This specimen is, however, much larger than any formally described *Sporogonites* species. Further taxonomic discussions are nonetheless deferred on account of the mediocre preservation of the single specimen.

The presence in both this specimen and *Sporogonites* sp. A of a small hemispherical structure terminating the sporangium is significant. A similar structure was observed by Halle^37^ on the *Sporogonites* (*Sporogonites exuberans*) type material from Röragen. In the most complete specimens, the tip of the sporangia is described as rounded but with a little break differentiating darker material around the extremity of the sporangium. Alternately this structure may be absent, and the sporangial tip characterised by a depression. Cyrille Prestianni (CP) has confirmed presence of this structure in specimens of *Sporogonites exuberans* from Belgium. From the perspective of the common identification of *Sporogonites,* as a bryophyte, this recurring structure likely represents the operculum of the capsule.

### Polysporangiophyta

^23^

*Cooksonia*^32^

*Cooksonia* *paranensis*^2^ .

**Figure 4:**
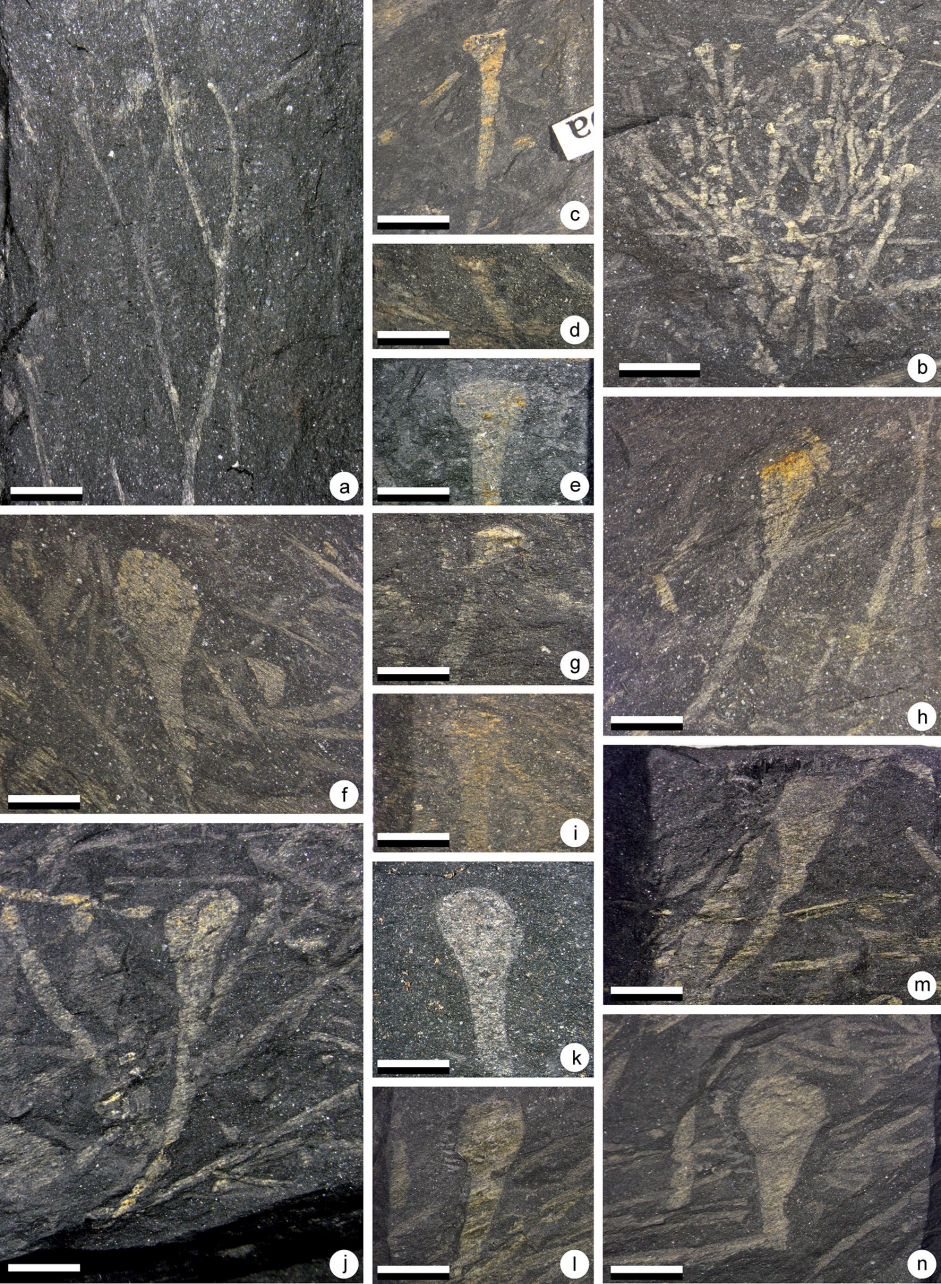
(**a**–**n**) *Cooksonia paranensis*^2,4^. (**a**) Specimen AM 7908. Scale = 1 cm. specimen showing three branching orders and a terminal sporangium. (**b**) Specimen AM 7906. Scale = 1 cm. Truss of the plant showing a number of branching orders and terminal sporangia. (**c**) Specimen AM 7910a. Scale = 5 mm. Sporangium. (**d**) Specimen AM 7887. Scale = 5 mm. Sporangium. (**e**) Specimen AM 7913. Scale = 5 mm. Sporangium. (**f**) Specimen AM 7912a. Scale = 5 mm. Sporangium. (**g**) Specimen AM 7888. Scale = 5 mm. Sporangium. (**h**) Specimen AM 7950b. Scale = 5 mm. Sporangium. (**i**) Specimen AM 7964. Scale = 5 mm. Sporangium. (**j**) Specimen AM 7958a. Scale = 5 mm. Sporangium. (**k**) Specimen AM 7965. Scale = 5 mm. Sporangium. (**l**) Specimen AM 7955a. Scale = 5 mm. Sporangium. (**m**) Specimen AM 7959a. Scale = 5 mm. Sporangium. (**n**) Specimen AM 7954b. Scale = 5 mm. Sporangium.

Fig. 4a–n; Fig. 7c and f

### Material

This plant is the most abundant in the UPL. It occurs either as isolated sporangia, sporangia connected to small stem fragments or as bunches of fertile axes. By contrast only two specimens have been identified in the LPL.

### Description

**Figure 5:**
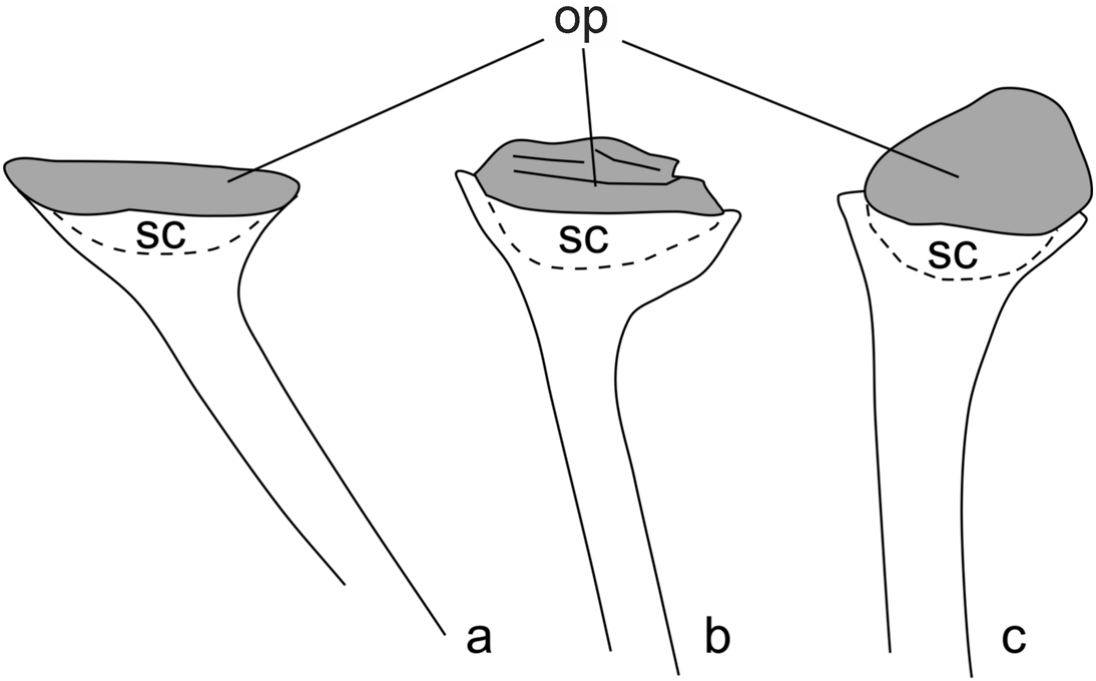
Schematic reconstruction of the sporangia of *Cooksonia paranensis* showing different position of the operculum (op) in regard to the sporangial chamber (sc): (**a**) in growth position with the operculum in place, (**b**) with the operculum compressed laterally showing several wrinkles and (**c**) with operculum completely tilted.

Several specimens of this plant have been discovered (Figs. 4, 7c,f). They consist of isotomously branched axes, 0.7–1.2 mm in width. In many cases, it is difficult to distinguish individual branching systems as, when not fragmentary, plants occur in bunches (Fig. 4b). This is particularly the case on one specimen that shows several isotomously branched plants arising from the same point (Fig. 4b). The specimens in Fig. 4a,b show the ultimate branching orders with sporangia attached. Branching systems always bear terminal sporangia (Fig. 4). Sporangia are trumpet- to cup-shape in outline and measure 2.5–5.0 mm in diameter and 2.0–3.0 mm in height. The axis/sporangium transition is progressive (Fig. 4c–m). It is thus difficult to identify with precision the base of the sporangium. The sporangial cavity gives the impression of being sunken into the subtending axis (Fig. 4f–k). The upper part of the sporangium is flat and marked by the presence of an apical plateau. The shape of the apical plateau seems very variable, but it results from differences in compression orientation (Fig. 5). In most cases, it is folded up, which results in a bulge (Figs. 5a, 4c–e) or in wrinkles (Figs. 5b, 4g). Several specimens seem to present a more spherical structure rather than an apical plateau (Fig. 4f,j–n). The apparent rounded shape of the sporangia is the result of the tilting of the apical plateau (Fig. 5c). This configuration was already noted by Gonez and Gerrienne^7^.

### Identity and comparisons

Plants with smooth isotomously branched axes, gradually widening distally into a single, terminal, cup- or trumpet shaped sporangium with an apical plateau can be attributed either to the genus *Cooksonia* Lang emend^6^ or to the genus *Concavatheca*^52^.

The genus *Cooksonia* was originally described by Lang^32^ on the basis of compression fossils exhibiting “dichotomously branched, slender, leafless stems, with terminal sporangia that are short and wide [with an] epidermis composed of elongate, pointed, thick-walled cells [and a] central vascular cylinder consisting of annular tracheids”. Thanks to the works of Edwards and collaborators (see among others:^35,36,7,9^), the genus is now known in great detail, on the basis of both compression and coalified specimens. The type species *C. pertoni* is considered the earliest eutracheophyte^23^. The genus diagnosis was emended in 2010^6^ at which time it included three well defined species: *C. pertoni*, *C. paranensis* and *C. banksii*^6^, however *C. banksii* has later been transferred to another genus (see below and Morris et al.^52^). *C. pertoni* and *C. paranensis* are morphologically similar, but, according to Gonez and Gerrienne^6^, *C. paranensis* can be distinguished from *C. pertoni* by its slender axes and the more gradual transition between axis and sporangium. As a result of this gradual transition, the sporangial cavity of *C. paranensis* is sunken in the subtending axis. The genus *Cooksonia* also includes three less well-preserved species, *C. hemisphaerica*^32^, *C. cambrensis*^35^ and *C. bohemica*^38^. All of these are considered doubtful by Gonez and Gerrienne^6^ because they are based on poorly preserved specimens. A restudy of the fossil material of *C. bohemica* has led Kraft et al.^41^ to place this plant within the genus *Aberlemnia* under the combination *A. bohemica.* Recently, a new species, *C. barrandei*^64^ has been described. The plant is morphologically close to *C. pertoni* and *C. paranensis*, but with more robust axes and bigger sporangia^64^.

The specimens originally described under the binomial *Cooksonia banksii* by Habgood et al.^7^ have been transferred to the genus *Concavatheca* by Morris et al.^52^. The genus *Concavatheca* includes plants with smooth axes and single terminal sporangia. The subtending axis gradually widens distally into a cup-shaped, sunken sporangial cavity. The specimens of *Concavatheca banksii* differ from those of *Cooksonia. pertoni* because the spore mass of *Concavatheca banksii* is sunken in the subtending axis whereas *Cooksonia pertoni* has a discoidal spore mass subtended by an axis that gradually increases in diameter. Other differences exist, both in sporangial structure and spore ultrastructure. In having a sunken sporangial cavity, the *species Concavatheca banksii* is very similar to *Cooksonia paranensis* and the two species are difficult to distinguish. They are mainly differentiated by their preservation type: compression for *C. paranensis* and charcoalification for *Concavatheca banksii.* Accordingly, detailed comparisons are not possible, and the two species can be kept in separate genera, on the basis of art. 11.1 of the International Code of Nomenclature for algae, fungi, and plants (Shenzhen Code)^18^, which states that “the use of separate names is allowed for fossil-taxa that represent different parts, life-history stages, or preservational states of what may have been a single organismal taxon or even a single individual”^18^, art. 11.1).

The specimens here described are morphologically very close to *Cooksonia pertoni*, *Cooksonia paranensis* and *Concavatheca banksii.* The very gradual transition between the sporangia and the subtending axes as well as the “sunken” aspect of the sporangial cavity are reminiscent of both *C. paranensis* and *Concavatheca banksii*. As our material, like previously described material of Cooksonia paranensis, is preserved as compression fossils it cannot be accurately compared with *Concavatheca banksia*, and we accordingly choose to name it *Cooksonia paranensis*.

### Age and distribution

*Cooksonia paranensis* was first described from the Ponta Grossa Formation at Jackson de Figueiredo (southern Paraná Basin, Brazil) as well as in four other localities of the Paraná Basin^2^. A Lochkovian age has been proposed for these plant bearing beds based on palynology^2^. One single other putative occurrence has been recorded. In the Lochkovian Talacasto Formation, at Talacasto creek (Argentina) Edwards et al.^43^ report one poorly preserved specimen that could be assigned with doubts to either *Cooksonia paranensis* or *Concavatheca banksii*.

*Cooksonia* hemisphaerica^32^

Fig. 6a–b.

**Figure 6:**
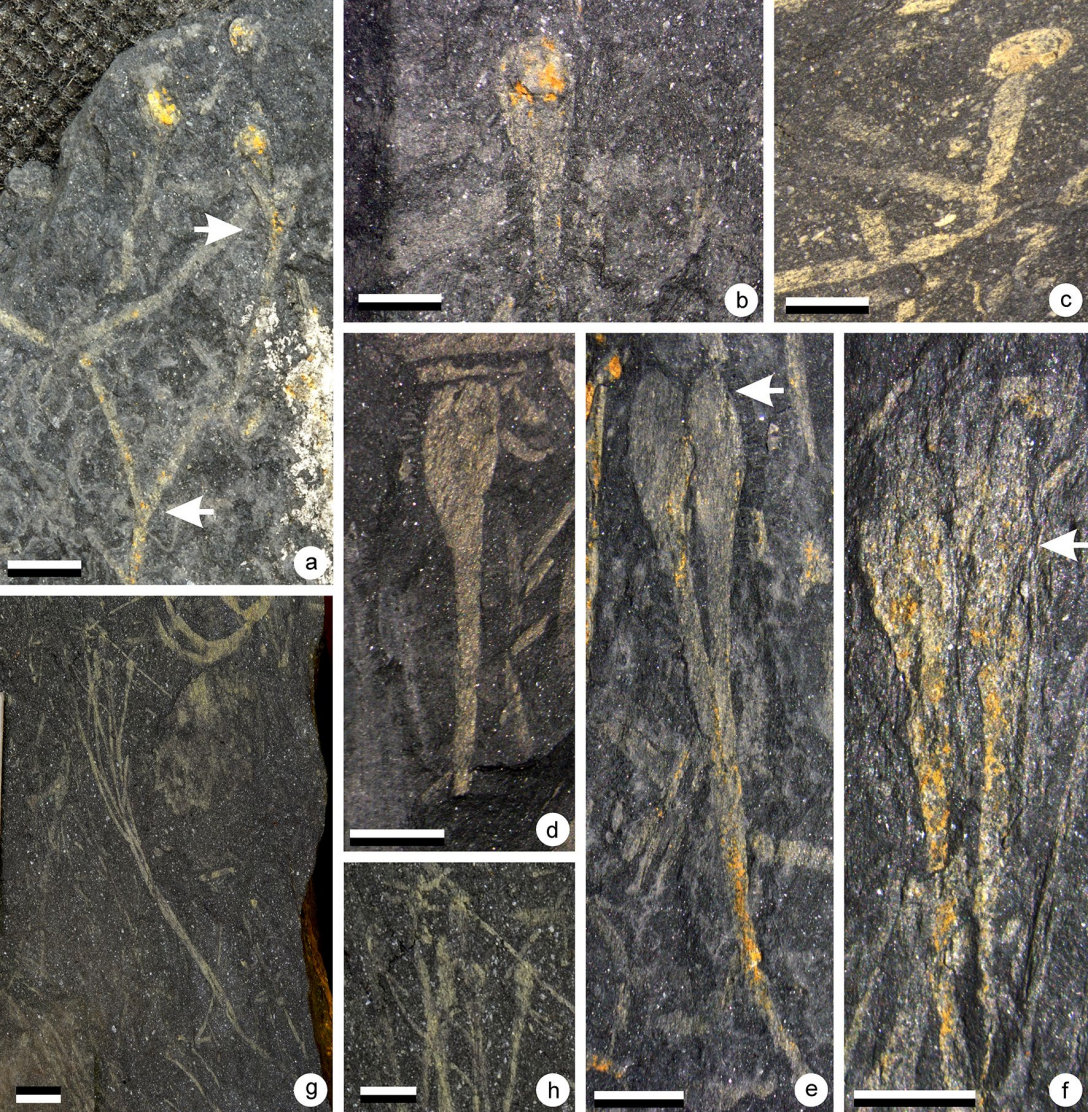
(**a,b**) *Cooksonia hemisphaerica,* (**c**) *Cooksonia cambrensis* Edwards^35^, (**d**–**f**) *Tortillicaulis* sp., (**g**–**h**) cf. *Cooksonia hemisphaerica,* (**a**) Specimen AM 7914. Scale = 1 cm. Specimen showing the gross morphology of the plant. (**b**) Specimen AM 7914. Scale = 5 mm. Detail of a rounded sporangium that is clearly distinct from the widening subtending axis. (**c**) Specimen AM5828. Scale = 2 mm. Sporangium and subtending axis. (**d**) Specimen AM 7956a. Scale = 5 mm. Specimen showing the organisation of the sporangia. An oblique striation is visible at the surface of the sporangium. (**e**) Specimen AM 7943. Scale = 5 mm. Specimen showing naked axes bifurcating one time before terminating in elongate sporangia. Note the oblique striation at the surface of the sporangia. (**f**) Specimen AM 7921a. Scale = 5 mm. A dichotomizing naked axis terminally bearing two sporangia. The sporangia bear a distinct oblique ornamentation and are made of two halves that are twisting on each other. (**g**) Specimen AM 7915a. Scale bar = 1 cm. Specimen showing the gross morphology of the plant. (**h**) Specimen AM 7925. Scale bar = 5 mm. Detail of specimen Fig. 6g showing the organisation of the sporangia.

**Figure 7:**
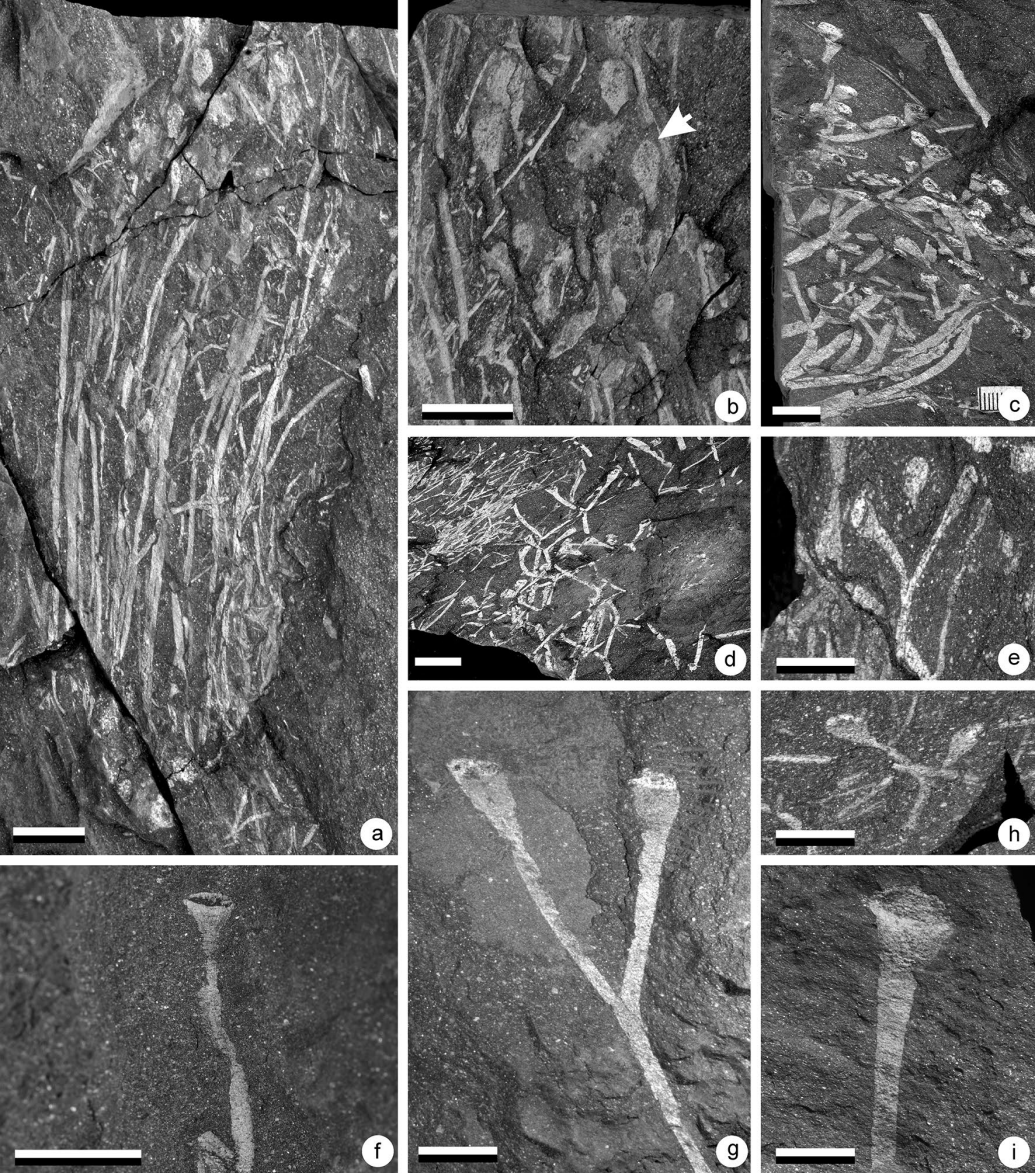
(**a**,**b**) *Sporogonites* sp. A, (**c**) and (**f**) *Cooksonia paranensis*, (**d**–**e**) and (**g**–**h**) *Steganotheca striata.* (**a**) Specimen AM 7927. Scale = 1 cm. A truss of non-branching more or less parallelly oriented axes distally bearing one single sporangium. (**b**) Specimen AM 7927. Scale = 5 mm. Detail of the distal ends and of the sporangia. The arrow point towards the limits of the small hemispheric structure present at the tip of the sporangium. (**c**) Specimen AM 7891a. Scale = 1 cm. Gross morphology of the specimen showing several superimposed individuals. (**d**) Specimen AM 7893. Scale = 1 cm. Gross morphology of the specimen showing several intertwined axes. (**e**) Specimen AM 7997. Scale = 5 mm. Isolated termination showing the ultimate dichotomy and two sporangia. (**f**) Specimen AM 7886. Scale = 5 mm. Detail of an isolated sporangium. (**g**) Specimen AM 7987. Scale = 5 mm. Distal part of the plant showing two more or less parallel sided sporangia. (**h**) Specimen AM 7998. Scale = 5 mm. Distal part of the plant showing two more or less parallel sided sporangia. (**i**) Specimen AM 7973. Scale = 5 mm. Isolated specimen showing the organisation of the sporangium.

### Material

One single moderately preserved specimen from the UPL.

### Description

The specimen consists of a 58 mm long dichotomous branching system terminally bearing sporangia (Fig. 6a). Two isotomous dichotomies are observed (see arrows on Fig. 6a). The first order axis is broken at its base and measures 20 mm long and 1 mm wide. Second order axes are 10–12.5 mm long and 0.8–1 mm wide. Finally, third order axes are short and measure 5 mm long and 1 mm wide.

Sporangia are globular, they measure between 1.3–2.0 mm long and 2.0–2.4 mm wide. Within the sporangium, a circular structure can be observed that we interpret as the sporangial cavity (Fig. 6b). This cavity measures between 1.5 and 1.8 mm in diameter. Sporangial wall is 0.3–0.4 mm thick. No dehiscence line could be observed.

The subtending axis is 2.9–3.6 mm long. It gradually flares and distally reaches 2.0–2.4 mm in width. The sporangium-axis contact is relatively large as the axis is almost as wide as the sporangium (Fig. 6b).

### Identity and comparison

Dichotomous branching systems distally bearing elongate structures are relatively common in the Lower Devonian. Identification is made difficult by the scarcity of morphological traits. In addition to *Cooksonia hemispherica*, four taxa are known to show such organisation. They are: *Tortilicaulis* Edwards^35^, *Tarrantia* Fanning et al.^36^, *Salopella* Edwards and Richardson^44^ and *Uskiella* Shute and Edwards^12^. They however all present an elongated sporangial cavity and a sharp transition at the sporangium-axis contact. The occurrence of isotomously branched axes distally bearing globular sporangia at the end of gently tapering subtending axes conforms to *Cooksonia hemisphaerica* Lang^32,35,36^. The here recorded size range conforms to the larger forms recorded from the British Isles.

*Age and distribution. Cooksonia hemisphaerica* was originally described from the Targrove quarry^32^. The Targrove quarry deposits have been dated by means of fishes and spores and are Lochkovian in age^36^. Later, Edwards^35^ reported specimens of *Cooksonia hemisphaerica* from Freshwater East in the South Dyfed region (South Wales). This occurrence has been dated through palynology and is considered Pridoli in age. Another occurrence is the Lochkovian Brown Clee Hill locality (Shropshire-England)^46^. *Cooksonia hemisphaerica* has also been recorded in the Bryn Glas borehole (Anglo-Welsh Basin)^42^.

Cf. *Cooksonia hemisphaerica*^32^

Fig. 6g–h

### Material

This plant is only known from a single more or less complete specimen collected from UPL.

### Description

It is characterized by a dichotomous branching system distally bearing globular sporangia (Fig. 6g–h). It is 130 mm long and consists of a three-times isotomously dichotomizing axis. The branching angles are small measuring less than 10°. First order axis is proximally incomplete and measures 35 mm long and 1.9 mm in width. Second order axes are 49 mm in length and 1.0 mm in width. The third order axes are 25–27 mm in length and 0.8–0.9 mm in width. The fourth ultimate order axes are 16–19 mm in length and 0.6–0.8 mm in width. Each third order axis bears one sporangium. The subtending axes of the sporangia gradually flare at a length of 6.0–7.0 mm to approximately reach the width of the sporangia (Fig. 6h). The sporangia are globular and measure 1.0–1.5 mm long. When measured at their widest point, sporangia are 2.0–2.2 mm in width.

### Identity and comparisons

Despite notable differences, the shape of the Impofu Dam specimens is most reminiscent of *Cooksonia hemisphaerica*. In this plant, sporangia are globose and borne on axes strongly widening just below them^32,36^. The recorded size range for the sporangia in *C. hemisphaerica* is compatible with the Impofu Dam material. However, *C. hemisphaerica* differs in that its sporangia are produced shortly after the ultimate dichotomy. Moreover, the identity of the “rounded tip” as a sporangium is equivocal as no clear division from the axis is visible. Therefore, we cannot unequivocally assign this material to *C*. *hemisphaerica* and so name the fossil cf. *Cooksonia hemisphaerica.*

*Cooksonia cambrensis*^35^

Fig. 6c

### Material

One single isolated sporangium from the UPL.

### Description

Sporangium born singly at the end of an unbranched smooth axis (Fig. 6c). The axis is 18 mm long and 0.7 mm wide. It slightly tappers distally to reach 1 mm at the base of the sporangium. The sporangium-axis junction is clear and flat. The sporangium is elliptical in outline, but we here suspect some deformation to have occurred. It measures 2.8 mm wide and 1.4 mm high.

### Identity and comparison

Although occurring as a single isolated specimen, it remarkably conforms to *Cooksonia cambrensis* to which we assign it based on the shape of the sporangium and the very limited tapering of the subtending axis^35,36^.

### Age and distribution

*Cooksonia cambrensis* has been identified in the Pridoli of Wales^35,47^ and the Lochkovian of England^36,47^.

*Steganotheca*^48^

*Steganotheca striata*^48^

Fig. 7d–e; 7 g–h.

### Material

This plant has exclusively been found in the LPL. Four specimens have been recovered occurring either as isolated stem fragments or as a relatively densely occurring plant mat.

### Description

Only the ultimate one or two branching orders have been recovered, up to 35 mm in overall length. The axes are isotomously branched (Fig. 7d–e, g–h). Their width is relatively constant throughout specimens and range from 0.6–1.4 basally to 0.8–1.4 mm distally. The axial surface is smooth. The sporangia are borne singly. They measure 2.9–4.2 mm long and 1.9–3.0 mm wide. The subtending axis widens rapidly at the transition to the sporangium. The width of the sporangium then remains constant for most of its length. The tip of the sporangium is truncated and seems to be topped by a denser looking lens-shaped apical plateau. The whole structure has the shape of a mug.

### Identity and comparison

The occurrence of smooth isotomous branching systems bearing isolated sporangia characterized by a flaring of the axes and topped by an apical plateau is indicative of either the genera *Cooksonia*^32^ or *Steganotheca*^48^. They chiefly differ in the shape of the sporangia. The occurrence of mug-shape terminal sporangia, that are longer than wide, parallel sided and truncated at the apex conforms to the genus *Steganotheca*. Despite the lack of minute details such as the striation observed on the sporangia of the original material from South Wales, we consider the similarities sufficient to attribute the Impofu dam material to the species *Steganotheca striata.*

### Age and distribution

*Steganotheca striata* has been recorded in the Silurian (Ludfordian and Pridoli) of South Wales (Capel Horeb Quarry)^48,49^.

*Aberlemnia*^5^

*Aberlemnia caledonica* (Edwards)^5^

Fig. 8a–d

**Figure 8:**
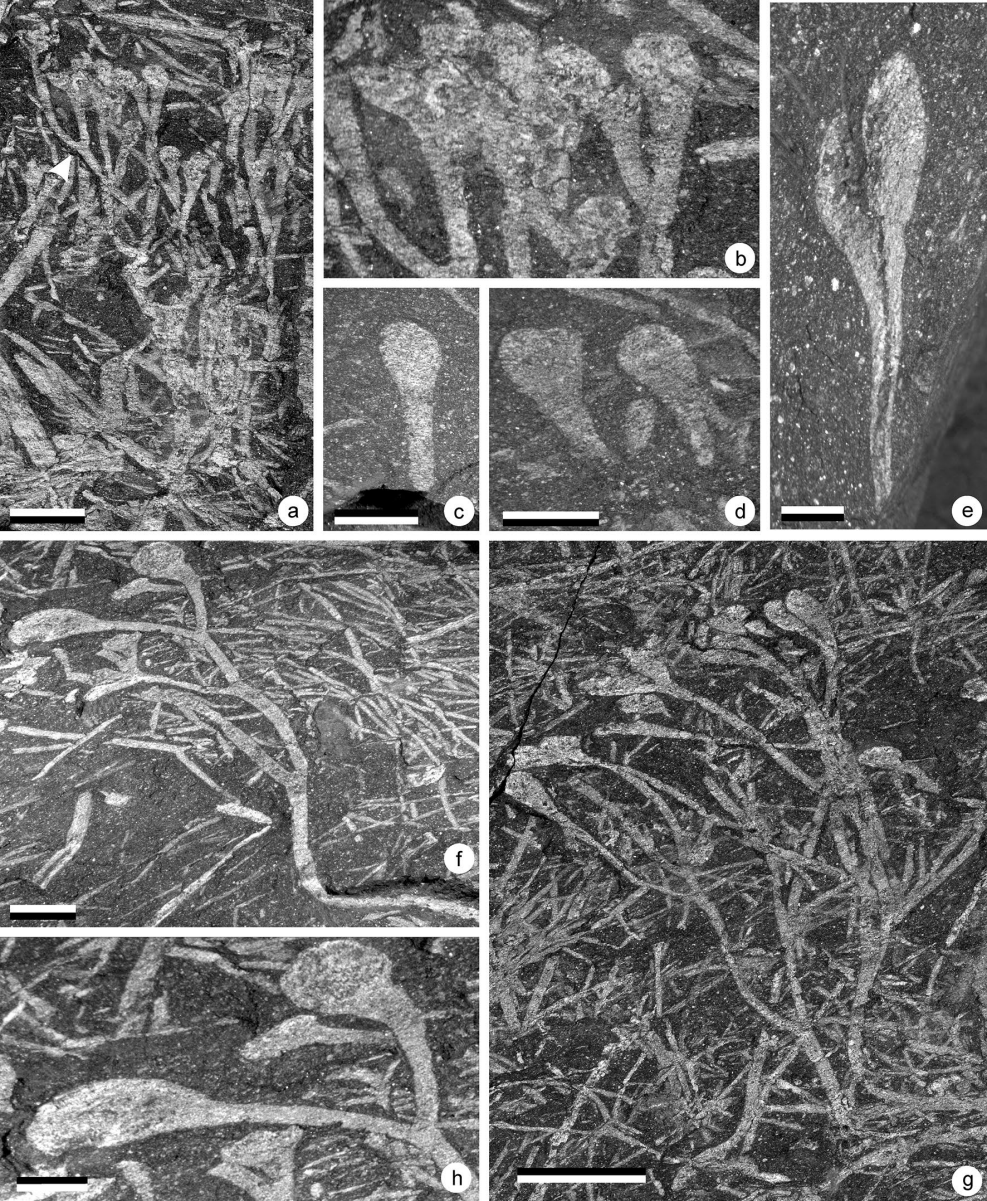
(**a**–**d**) *Aberlemnia caledonica*, (**e**) *Tortilicaulis* sp.*,* (**f**–**h**) *Uskiella spargens*. (**a**) Specimen AM 7970. Scale = 1 cm. Gross morphology of the plant showing its dense organisation. (**b**) Specimen AM 7970. Scale = 5 mm. Detail showing the organisation of the sporangia. (**c**) Specimen AM 7985. Scale = 5 mm. Detail of a subcircular sporangium. (**d**) Specimen AM 7980. Scale = 5 mm. Detail several subcircular sporangia. (**e**) Specimen AM 7984a. Scale = 5 mm. Ultimate dichotomy of the plant bearing two sporangia. Note the occurrence of an oblique striation at the surface of the sporangia. (**f**) Specimen AM 7966a. Scale = 1 cm. Isolated specimen showing the gross morphology of the plant. Note the horizontal axis and the marked curvature. (**g**) Specimen AM 7968a. Scale = 1 cm. Specimen showing the gross morphology of the plant. (**h**) Detail of specimen in fig. 7f showing two sporangia. Scale = 0.5 cm.

### Material

Four specimens including a more or less complete branching system have been recovered from the LPL.

### Description

The largest specimen of this plant is figured in Fig. 8a. It is relatively densely packed and therefore it is difficult to describe individual branching systems. It consists of branched axes terminated by reniform to transversely elongated sporangia (Fig. 8a,b). The axes are smooth and mostly dichotomize isotomously. They are, however, some indications of anisotomous division, but preservational limitations precludes any definite statement thereon (Fig. 8a). Axial width is constant throughout specimens and measure 0.5–0.7 mm. Branch length decreases distally. The ultimate division gives rise to short axes. Sporangia are sub-circular in outline and measure 1.0–1.8 mm in width and 0.9–1.6 mm in length (Fig. 8b–d). Subtending axes widen sharply just beneath sporangia and reveal a curved axis-sporangium junction.

### Identity and comparison

The occurrence of sub-circular terminal sporangia on mostly isotomously dichotomizing axes points towards the genera *Aberlemnia* and *Sporathylacium.* The prior was erected by Gonez and Gerrienne^5^ in order to accommodate specimens with reniform sporangia formerly included in the genus *Cooksonia.* The latter was established by Edwards et al.^50^ for bivalved reniform anatomically preserved sporangia. In the absence of any evidence for bivalved sporangia and considering the lack of anatomical details and of in situ spores in the Impofu dam material, we assign this material to *Aberlemnia caledonica*.

### Age and distribution

*Aberlemnia* has been recorded from the Lochkovian of Scotland^48^, the late Silurian to Lochkovian of Wales^35,36,47,49^, the Lochkovian of Brazil^2^, and possibly from the Ludlow of Bolivia^51,74^. Recently, Kraft et al.^41^ have suggested that the Late Silurian *Cooksonia bohemica* should be reassigned to the genus *Aberlemnia.* However, further studies are necessary to ascertain validity of this proposition.

*Tortilicaulis*^35^

*Tortilicaulis* sp.

Fig. 6d–f; Fig. 8e;

### Material

Specimens of this plant consists of 3 specimens from the UPL and one specimen from the LPL.

### Description

In all cases only one (Fig. 6d) or two (Figs. 6e–f, 8e) ultimate branching orders have been preserved. The preserved plant comprises large elongate sporangia terminating dichotomous axes. Sporangia are fusiform and taper distally to a blunt tip. The tip is obscured in specimen Fig. 6d by the occurrence of a transversely oriented axis. The sporangia show a clear twisted organization (see arrows on Fig. 6e,f). An obliquely oriented striation is observable on the surface of most sporangia. The measured angle of this striation is relatively variable and measures 24° (Fig. 6e), 28° (Fig. 6d) and 38° (Fig. 8e) from the vertical. A similarly oriented longitudinal splitting here interpreted as a dehiscence line is repeatedly observed (Figs. 6e–f, 8e). This is particularly visible on specimen Fig. 6f. This specimen is dehisced, and the putative two valves of the sporangia are slightly separated and twisted around each other. The junction with the subtending axis is unclear except on Fig. 6f where it is marked by the beginning of the putative dehiscence line. Maximum width of the sporangia occurs at mid-height. They measure 1.2–1.4 mm long and 0.3–0.4 mm wide in the UPL and 0.6 mm long and 0.2 mm wide in the LPL. The height to width ratio ranges between 3 and 3.5. The subtending axes are parallel sided but a faint widening upwards can be seen. Obliquely oriented cellular patterns could be observed on the subtending axes. Dichotomies are largely isotomous, branching at an acute angle between 10° and 15°.

### Identity and comparison

The occurrence of relatively large fusiform sporangia terminating isodichotomously branched smooth axes with relatively low branching angles, points towards three genera, namely *Salopella* Edwards and Richardson^44^,* Tortilicaulis*^35^ and *Teruelia*^53^. *Salopella* is characterized by longitudinally aligned elongated cells on the sporangia whereas these are obliquely oriented in *Tortilicaulis* and *Teruelia*. The latter however differs in being characterized by a multi-slit dehiscence whereas *Tortilicaulis* exhibits only one dehiscence slit. The oblique striation and the single dehiscence slit observed on the Impofu dam sporangia therefore support assignment to the genus *Tortilicaulis*. The only two species included in this genus are *T. transwalliensis* and *T. offaeus*. The Impofu dam material all falls within the size range of *T. transwalliensis* as described from the Targrove quarry^36^*.* The height to width ratio is however slightly smaller in the South African material but falls within the range of *T. transwalliensis* in general^35,36^. *Tortilicaulis transwalliensis* includes a very large size range from very small to relatively large forms. Size and proportion of the sporangia therefore appear to provide a relatively weak taxonomic guideline. Besides the characteristics of the sporangia, the main difference between the Impofu dam material and previously described *T. transwalliensis* is the presence of a very long subtending axis. Although, morphologically very close to *T. transwalliensis* we cannot unequivocally assign our new material to this species and so assign the fossil to *Tortilicaulis* sp.

### Age and distribution

The genus *Tortilicaulis* was first described from the Pridoli of South Wales^35^. The species *T. transwalliensis* has further been reported from the Ludlow Targrove Quarry (Shropshire, UK)^36^ and from the Lochkovian Bryn Glas Borehole (South Wale, UK)^42^. *Tortilicaulis offaeus* has been described from the Lochkovian of North Brown Clee Hill locality (Shropshire, UK)^46^. *Tortilicaulis* cf. *offaeus* from the Lochkovian Tredomen Quarry (South Wales, UK). Another hypothetical record comes from the Lower Devonian (Lochkovian?) Argentinian Villavicencio Formation with the record of a cf. *Tortilicaulis*^54^.

*Uskiella*^12^

*Uskiella spargens*^12^

Fig. 8f–h

### Material

Seven specimens were recovered from the LPL.

### Description

This plant consists of isodichotomous naked axes terminated by ovate structures. We assume these structures to be sporangia even though no in situ spores were observed.

One exceptionally large specimen, AM7968, is 51 mm long and consists of an axis that branches six-times (Fig. 8g). The first order axis is broken and measures 1.3 mm in diameter. It is considered to have been horizontal. After the first dichotomy, it gives rise to two axes that curve upwards 6 and 7 mm following an angle of 91° and 94°. They both measure 14 mm long and 1.3 mm wide. Each of these further dichotomizes at most three more times. The branching pattern is obscured by the density of the branching. All divisions appear to occur isodichotomously even though a slight anisotomy is possible. Third order axes are 9–13 mm long and 0.7–0.8 mm wide; fourth order axes are unclear but measure between 3 and 7 mm long and 0.6–0.8 mm wide; fifth order axes are 5–8 mm long and 0.6–0.8 mm wide. The sixth order axes constitute the ultimate one and each terminate in a sporangium. These are very short and measure 1.2–3.5 mm long and 0.6–0.8 mm wide. The gradual axis/sporangium transition makes precise measurement of the sporangium and its subtending axis difficult. Thus, we arbitrarily considered the place where the subtending axis is no longer parallel sided to mark the base of the sporangium. The sporangia are ovate in shape. They are characterized by a widening of the subtending axis that gives to the whole structure the shape of a tennis racket. They measure 3.4–4.7 mm long and 1.3–2.3 mm wide. No specialized structure for dehiscence is observed. The outline of the sporangia is very variable. The axis/sporangium junction is difficult to observe with precision but is marked by a slightly convex to almost straight line.

Another large specimen is AM7966 (Fig. 8f). It is 45 mm long and consists of an axis that branches three-times. The first order axis is 25 mm long and is presumed to have been horizontal for the first 14 mm, after which it is inflected by 108°. It measures 1.2 mm wide before the inflection and 1.4 thereafter. The plant subsequently dichotomizes three times. Second order axes are 9.5–10.8 mm long and 12–1.3 mm wide; third order axes are 5.4–6.8 mm long and 0.9–1.0 mm wide; fourth order axes are 4.1–5.9 mm long and 0.7–0.8 mm wide. The ultimate order axes measure 1.8–2.5 mm long and 1.0–1.2 mm wide. Only two sporangia are sufficiently well preserved to be accurately studied (Fig. 8h). They are rounded to ovate in shape and measure 6.2 and 3.4 mm long and 2.9 and 3.9 mm wide respectively. As with the previous specimen the axis/sporangium junction is marked by a slightly convex to almost straight line.

In summary, this plant seems to be characterized by a horizontal axis that dichotomizes at least once before curving at an angle approaching 90° and giving rise to what we interpret as the erect part of the plant. It subsequently dichotomizes three more times. Dichotomies seem to occur more or less isotomously but branching is relatively difficult to interpret in both specimens. The sporangia are tennis-racket shaped in outline..

### Identity and comparisons

The presence of longitudinally elongated sporangia showing a tapering base is initially suggestive of the genus *Salopella* Edwards and Richardson^44^. However, this genus differs significantly in shape as it is consistently described as having sporangia with acute tips and tapering apices^44,46^.

Rounded to elongate relatively large sporangia have repeatedly been reported in the literature^56^. The shape of these sporangia resembles two specimens originally described by Croft and Lang^56^ as *Cooksonia* sp. but later identified as cf. *Sporogonites* by Cookson^57^. They were both rediscussed by Shute and Edwards who highlighted the occurrence of a longitudinal slit on the sporangia which defined two valves. The material was therefore reassigned to the species *Uskiella spargens.* We believe that the Impofu Dam material is more or less identical to adpression material thereof from both Wales (UK) and Victoria (Australia). Evidence for the occurrence of two valves in our material is tenuous, however a double valved sporangium would explain the variability of the shape of the sporangia observed in several specimens (Fig. 8h). In addition, the Impofu Dam specimens fall within the same size range as *Uskiella spargens*. *Uskiella reticulata*^36^ is characterized by much smaller sporangia. Considering the many similarities existing between the Impofu Dam material and both the Australian and Welsh material and despite the lack of definite evidence of a longitudinal dehiscence slit, we attribute the here described material to *Uskiella spargens*.

### Remarks

One of the important features of this plant is the description of an extensive branching system. The occurrence of a dichotomizing horizontal axis giving rise to an erect plant has repeatedly been observed in plants of more or less the same age such as *Aglaophyton majus, Rhynia Gwynne-vaughannii* or *Nothia aphylla* (see Hetherington and Dolan for references). The lack of anatomical preservation in the Impofu Dam material precludes a rigorous interpretation of these axes. It is, however, hypothesised that these dichotomizing horizontal axes performed the sporophyte rooting function. This suggest that *Uskiella spargens* lacked a true rooting system.

### Age and distribution

*Uskiella spargens* was originally described from the Pragian of Wales^12^ Specimens identified as *Cooksonia* sp*.* by Croft and Lang^56^ and later synonymized with *U. spargens* were collected from the Lochkovian of Allt Du (South Wales)^59^. Specimens originally described as cf. *Sporogonites* but synonymized with *U. spargens* by Shute and Edwards^12^ were collected from the Lochkovian to lower Pragian Humevale Siltstone Formation of Lilydale (Australia)^57^.

### Genus

#### Krommia gen. nov.

**Type species:** *Krommia parvapilla* sp. nov.

**Derivation of the name:** *Krommia* from the Kromme River (from Afrikaans meaning curved).

**Diagnosis:** Plant with smooth, three dimensional, isotomously branching axes; Sporangia small and rounded borne singly and terminally.

### Species

#### Krommia parvapila sp. nov.

**Derivation of the name:** *parvapila*, from Latin a small ball referring to the sporangium.

**Diagnosis:** Same as for genus. Dichotomizing up to three times. Branching angle variable (40°–110°). First and second order axes U shaped. Axes 1.5–3.0 mm long and 0.3–0.35 mm wide below sporangia. Small constriction at junction between subtending axis and sporangium. Sporangia rounded between 0.7 and 0.8 mm in diameter.

**Figure 9:**
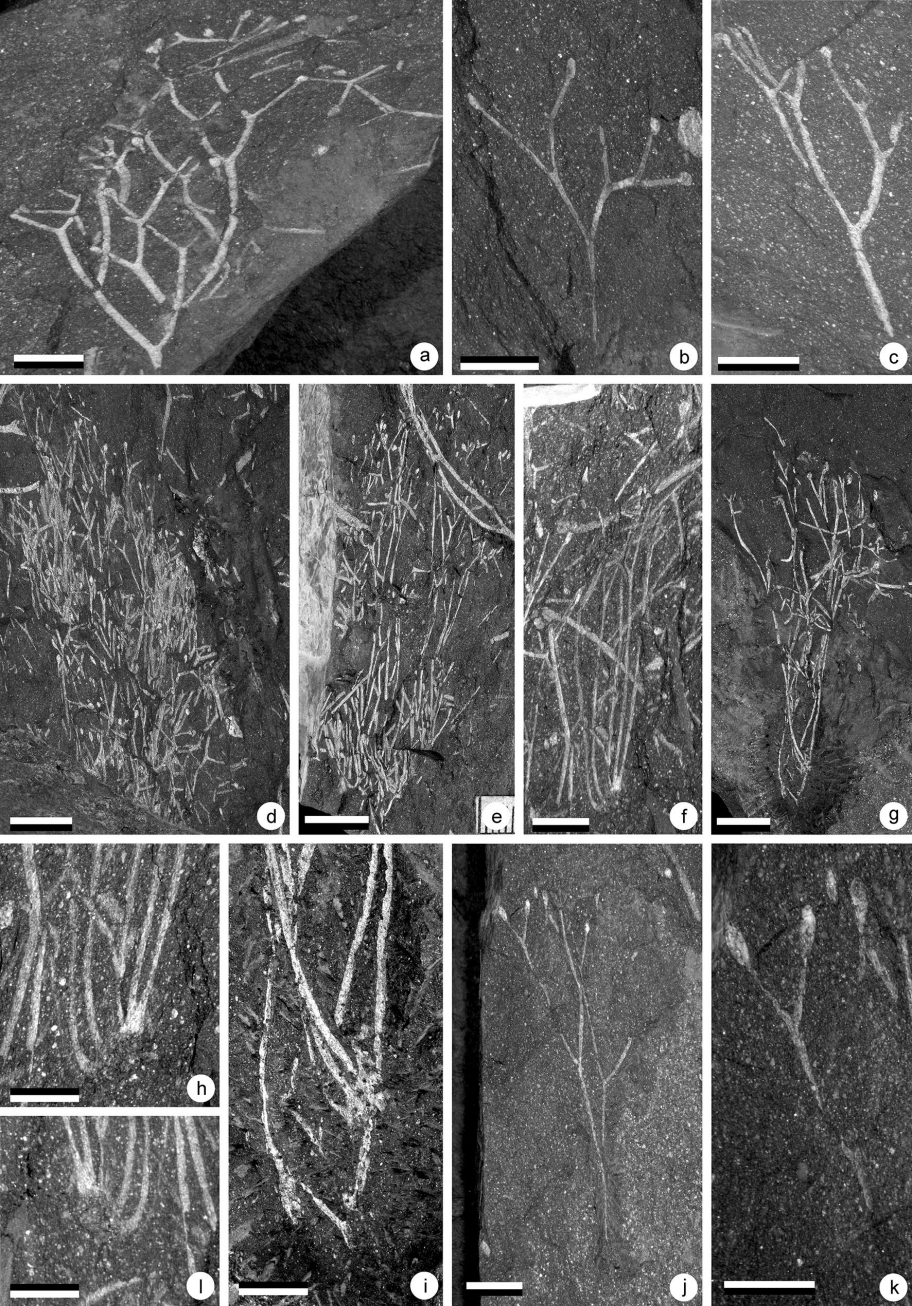
(**a**–**c**) *Krommia parvapilla* gen. nov. sp. nov., (**d**–**l**) *Elandia itshoba* gen. nov. sp. nov. (**a**) Specimen AM 7929. Scale = 5 mm. Gross morphology of the plant showing the U-shaped first and second order axes. (**b**) Specimen AM 7928a. Scale = 5 mm. Slightly laterally compressed specimen showing the different branching orders and the terminal rounded sporangia. (**c**) Specimen AM 7969b. Scale = 5 mm. Specimen the U-shaped first order axes and the terminal sporangia. (**d**) Specimen AM 7894a. Scale = 5 mm. (**e**) Specimen AM 7897a. Scale = 5 mm. (**f**) Specimen AM 7975a. Scale = 5 mm. (**g**) Specimen AM 7988a. Scale = 5 mm. (**h**) Specimen AM 7975b. Scale = 3 mm (**i**) Specimen AM 7988a. Scale =  4 mm(**j**) Specimen AM 7932a. Scale = 5 mm. (**k**) Specimen AM 7931a. Scale = 3mm (**l**) Specimen AM 7975a. Scale = 3 mm.

**Holotype**: AM 7928a (part) and AM 7928b (counterpart), Fig. 9a.

**Paratypes**: AM 7929 and AM 7969.

**Repository**: Albany Museum, Devonian Lab, Beaufort Street, Makhanda, Eastern Cape, South Africa.

**Type locality**: Impofu Dam, Kouga Municipality, Eastern Cape, South Africa (Fig. 1).

**Horizon**: Kareedouw Member, Baviaanskloof Formation, Nardouw Subgroup, Table Mountain Group, Cape Supergroup.

**Age**: Lower Devonian, Lochkovian?

**Synonymy**: *Minutia fragilis* nomen nullum Gonez^31^, Fig. 1 p. 178, from Jackson de Figueiredo, Jaguariaiva county, Brazil.

Fig. 9a–c

### Material

Five specimens of this plant have been recovered from the LPL.

### Description

In all specimens, only the ultimate parts of the plants have been preserved. They measure up to 20 mm in length and consist of smooth axes that branch isotomously at relatively variable angles (40–110°). This variability suggests that the branching system was three dimensional. We think that relatively wide angles (70–110°) were originally present and compressed during taphonomical processes. A maximum of three dichotomies has been observed however the occurrence on Fig. 9a of two superimposed similar branching systems in the same orientation suggests that more dichotomies can be expected. On this specimen only a small part of the first branching order has been preserved which measures 0.9 mm wide. It branches to produce two slightly different axes measuring 8–10 mm long and 0.6 and 0.7 mm wide respectively. The exact branching pattern is then obscured by the superimposition of at least one other branching system; however, it clearly branches two more times. The first and second branching orders are characterized by a slight curvature that give to the pair of axes a U rather than a V shape. The specimens are terminated by small rounded sporangia measuring 0.7–0.8 mm wide. The junction between the subtending axes and the sporangia is marked by a small constriction of the axis. Subtending axes measure 1.5–3.0 mm long and 0.3–0.35 mm wide.

A similar branching pattern is observed in specimens illustrated in Fig. 9b and c. First axes orders are incomplete and measure 6.5–7.5 mm long and 0.5–0.8 mm wide respectively. Second branching orders measure 3.8–4.8 mm long and 0.3–0.4 mm wide in Fig. 9b and 5.0–8.0 mm long and 0.6–0.7 mm wide in Fig. 9c. Third order branches measure 2.0–3.5 mm long and 0.3 and 0.5 mm wide in Fig. 9b and 2.5–3.5 mm long and 0.4–0.6 mm wide in Fig. 9c. The fourth order axes also are the subtending axes of the sporangia. They measure 2.4–2.8 mm long and 0.3–0.5 mm wide in Fig. 9b and 3.4–3.5 mm long and 0.3–0.4 mm wide in Fig. 9c. They always end in a small constriction that marks the base of the sporangia. The sporangia are rounded and measure 0.7–0.8 mm in diameter. No dehiscence feature was observed.

### Comparison

Small plant remains with minute (mesofossil sized) sporangia have repeatedly been observed in Silurian to Lochkovian deposits^60,62,11,46,73^. Occurring as isolated plant fragments most of them were kept in open taxonomy. Several plant fragments resembling the Impofu Dam material were illustrated by Morris et al. ^73^. Our specimens most closely resemble their morphotype C however the characteristic constriction at the base of the sporangia has not been reported. One of their illustrated specimens does however show a similar structure. Croft and Lang^56^ published several specimens as *Cooksonia* sp.. Mainly consisting of isolated sporangia, they could bear some superficial resemblance to the SA specimens however detailed comparison is made difficult by the absence of vegetative structures. Our specimens more closely resemble the more fragmentary Brazilian specimens illustrated in the unpublished thesis of Gonez^31^. They share the same overall organization including the curvature of the second and third order axes. The characteristic constriction at the base of the sporangia is also present and the sporangia are comparable in shape and size, suggesting that they represent the same species. The Brazilian material has however never been validly published. Considering the extensive branching system preserved and the occurrence of this plant both in Brazil and South Africa we chose to erect a new genus and species.

### Genus

#### Elandia gen. nov.

**Type species:** *Elandia itshoba* sp. nov*.*

**Derivation of the name**: after Eland (*Taurotragus oryx*), from Elandsjacht, original farm name of locality, meaning Eland hunt in Afrikaans.

**Diagnosis**: Plant forming dense trusses of fine, smooth, isotomously branching axes. Axes bifurcating at a low angle and terminating in minute, elongate ovate sporangia; multiple axes united by a basal structure.

### Species

#### Elandia itshoba sp. nov.

**Derivation of the name**: from isiXhosa, itshoba, a ritual fly whisk made from a bulls tail, sometimes with the hair tips decorated with tiny beads.

**Diagnosis:** As for genus, bifurcating up to four times, sporangia straight sided with broadly rounded apices. Sporangia 1.3–1.6 mm long and 0.4–0.7 mm wide.

**Holotype**: AM 7932a (part) and AM 7932b (counterpart), Fig. 9j.

**Paratype**: AM 7894, AM 7897, AM 7975, AM 7988.

**Repository**: Albany Museum, Devonian Lab, Beaufort Street, Makhanda, Eastern Cape, South Africa.

**Type locality**: Impofu Dam, Kouga Municipality, Eastern Cape, South Africa (Fig. 1).

**Horizon**: Kareedouw Member, Baviaanskloof Formation, Nardouw Subgroup, Table Mountain Group, Cape Supergroup.

**Age**: Lower Devonian, Lochkovian?

Fig. 9d–l

### Material

Seven specimens from the LPL.

### Description

This plant most often occurs as densely packed trusses of axes. This renders description of individual axes’ organization difficult. In all specimens, it consists of up to 60 mm long thin smooth axes that branch up to four times and bear minute terminally elongate ovate sporangia.

The organization of the plant is best seen in AM 7932a (Fig. 9j), which is proximally incomplete. It measures 33 mm long and branches 4 times. 7.2 mm of the first order axis is preserved which is 0.3 mm wide. Second order axes are 7.0 and 9.5 mm long and 0.2 and 0.3 mm wide respectively. Fourth order axes are 6.5–8 mm long and 0.2–0.3 mm wide. Only one fifth order axis is preserved and measure 4.5 mm long and 0.3 mm wide. The sixth order axes correspond to the subtending axes of the sporangia. They measure 2.4–3.1 mm long and 0.2–0.3 mm wide. The junction between the sporangium and the subtending axis is clear.

The base of the plant is best seen on Fig. 9f,g. When preserved, first order axes are long, only dichotomize after 18 to 19 mm and are 0.3 mm wide. Several more or less parallelly disposed axes converge basally on a poorly preserved structure of indefinite shape (Fig. 9h,l). Up to 6 axes are observed to arise from this structure that is here interpreted as a remnant of the gametophyte. It is up to 4 mm wide. Figure 9d and e however suggest that in life a larger number of axes were probably attached to a more extensive gametophyte or cluster of gametophytes.

The sporangia are vertically elongated and straight sided with broadly rounded apices. They measure 1.3–1.6 mm long and 0.4–0.7 mm wide.

### Identity and comparison

As discussed for *Krommia parvapilla*, the occurrence of very small sporangia has been reported many times, mostly from Wales. In the majority of cases however, they are found isolated or connected to very fragmentary branching systems. The very limited available information (mostly the shape of the sporangium) makes comparison and identification not only difficult but also very likely misleading. The extensive branching system preserved in the Impofu Dam material allows for a better description of the plant to be made. Three features are noticeable, the shape of the sporangia (elongated, parallel sided and rounded tips), the delicate branching system with relatively small branching angles and the occurrence of possible gametophytic tissues at the base of the plant. As already discussed above, even if the shape of the sporangia is by far the most informative character in early land plants, it is also misleading as they are very simple. When comparing the sporangia alone, the Impofu Dam material most closely resembles *Tarrantia salopensis* Fanning et al.^36^ and *Uskiella reticulata* Fanning et al.^36^*.* The sporangia are however distinct, being much smaller and presenting a height/width ratio of 3.3 which is much higher than that encountered in these two species. The sporangia further lack the characteristic reticulation of *U. reticulata*. When comparing the whole plant, the Impofu Dam material very closely resembles *Eogaspesia gracilis* Daber^75^. This plant has been described as small slender dichotomizing axes being borne on a thicker dichotomous rhizome and bearing small ovate sporangia. Based on the illustrations, the connection between the axes and the so-called rhizome are dubious. Despite superficial resemblance, the south African material differs from *Eogaspesia* by being smaller (80–90 mm long for *Eogaspesia* as opposed to 60 mm for *Elandia*) and by presenting isotomous divisions only. The sporangia though very simple and thus difficult to compare differ in shape being more elongate (length/width ration of 3.8 in *Elandia* as opposed to 2.5 in *Eogaspesia*) are more parallel sided and have more rounded tips. We therefore exclude the Impofu Dam material from these two taxa. As far as we know, the combination of characters described above has never been encountered before. We thus chose to erect a new genus and a new species.

### Genus

#### Mtshaelo gen. nov.

Type species: *Mtshaelo kougaensis sp. nov.*

**Derivation of the name**: isiXhosa, a traditional broom.

**Diagnosis**. Plant with multiple (at least 6) elongate sporangia, spindle-shaped in profile and evenly tapering to acute terminations, truncated proximally at point of attachment; arranged in a truss of sporangia that terminates elongate parallel sided isotomously bifurcating axes.

### Species

#### Mtshaelo kougaensis sp. nov.

**Diagnosis**. As for genus, robust axes bifurcate at least twice and widen slightly towards the terminal truss of sporangia; individual sporangia 0.74 to 0.8 mm wide and 4–6 mm long with a longitudinal dehiscence line. Vegetative axes 1.0 to 1.4 mm wide.

**Derivation of the name**: Kouga is the name of the district in which the site is found, from Khoisan meaning ‘place of plenty’, -ensis from Greek meaning from.

**Figure 10:**
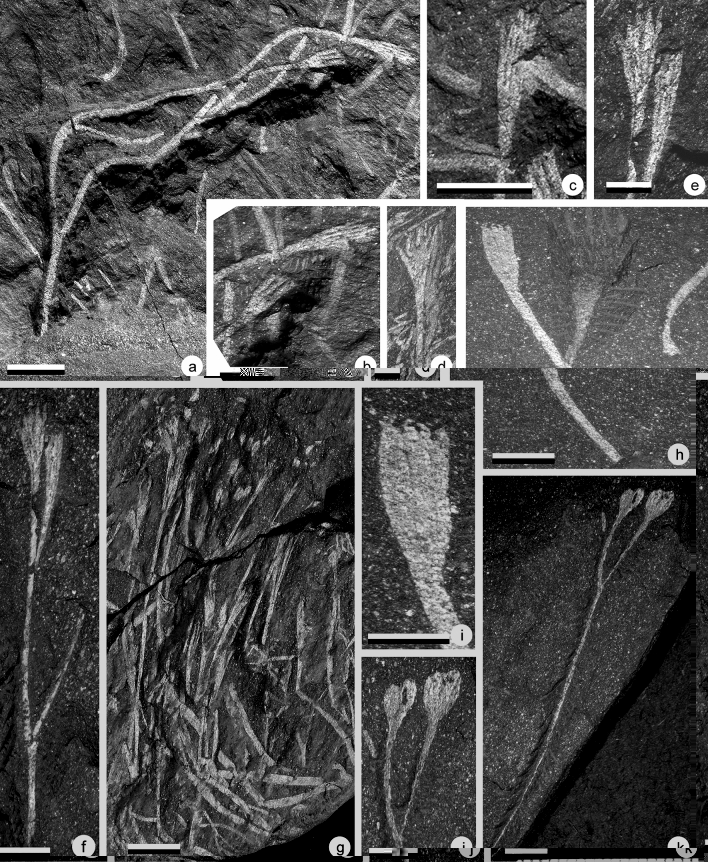
(**a**–**g**) *Mtshayelo kougaensis* gen. et sp. nov., (**h**–**i**) *Yarravia oblonga*, (**j**–**k**) incertae sedis bilobed sporangia. (**a**) AM 7999a. Scale = 1 cm. Holotype. Bifurcating naked axis terminated by two synangiate structures. (**b**) Detail of AM 7999a. scale = 5 mm. Two synangiate structures. (**c**) Detail of AM 7999a. Scale = 5 mm. Detail showing the organization of a synangiate structure with the dehiscence line clearly visible. (**d**) Am 7902. Scale = 5 mm. Isolated synangiate structure. (**e**) AM 7904. Scale = 2.5 mm. Isolated branching axis showing two dichotomies and two terminal synangiate structures. (**f**) Detail of AM 7904. Scale = 5 mm. Two terminal synagiate structure. The arrow indicates where individual sporangia are starting. (**g**) AM 7990. Scale = 1 cm. Specimen showing several superimposed plants. (**h**) AM 7983. Scale = 5 mm. Gross morphology of the plant. (**i**) Detail of AM 7983. Scale = 5 mm. Detail of the synangiate structure showing four closely adpressed individual sporangia with a pointed tip. (**j**) AM 7933. Scale = 1 cm. Gross morphology of the plant. (**k**) Detail of AM 7933. Scale = 5 mm. Sporangia.

**Holotype**: AM 7999a (part), Fig. 10a.

**Paratypes:** AM 7902, AM 7904, AM 7933, AM 7983, AM 7990.

**Repository**: Albany Museum, Devonian Lab, Beaufort Street, Makhanda, Eastern Cape, South Africa.

**Type locality**: Impofu Dam, Kouga Municipality, Eastern Cape, South Africa (Fig. 1).

**Horizon**: Kareedouw Member, Baviaanskloof Formation, Nardouw Subgroup, Table Mountain Group, Cape Supergroup.

**Age**: Lower Devonian, Lochkovian?

**Figure 11:**
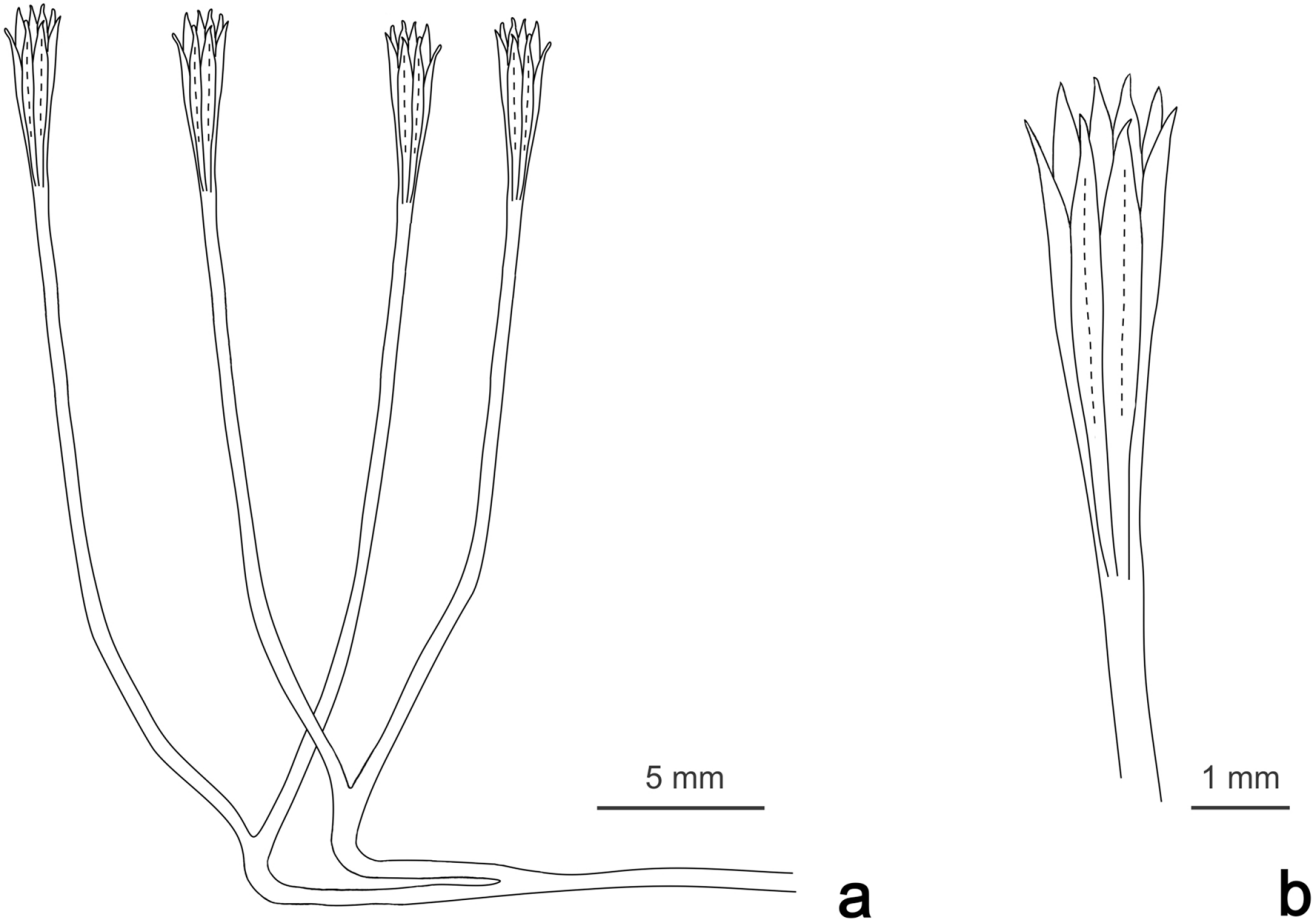
Proposed reconstruction of *Mtshaelo kougaensis* (**a**) and of its synangiate structure (**b**).

Fig. 10a–g, Fig. 11.

### Material

Five specimens of this plant have been collected from the LPL.

### Description

This plant most often occurs as isolated branched axes (Fig. 10a–f) but can in some cases occur as densely packed trusses of axes (Fig. 10g). In the latter the organization of the branching system is obscured by the many superimpositions. The plant consists of robust smooth parallel-sided axes that dichotomize up to at least two times and terminate in trusses of elongate structures. The lack of preserved anatomy or spore contents prevents demonstration of the fertile nature of these structures. Considering their position and in analogy with other plants of similar organization we will consider them as sporangia. All sporangia seem to be attached at the same level giving to the whole structure the aspect of a synangium.

The organization of the vegetative parts is best seen in specimen AM 7999 (Fig. 10a). This specimen is 80 mm long and has a generally flexuous appearance despite the robust aspect of the axes. It branches at least two times but only two synangium-like structures are preserved, only one of which exhibits clear attachment to the full branching system. The first order axis is 17 mm long and 1.4 mm wide. It branches at an angle of 32°. Second order axes are both quite flexuous and marked by a 90° curvature in the same direction. The left hand more completely preserved axis measures 20 mm long and 1.4 mm wide. It is difficult to say whether the evident curvature was originally present or the consequence of taphonomical processes. It could represent a horizontal part of the sporophyte as proposed in Fig. 4.

Only one third order axis is fully exposed. It measures 39 mm long and is1.2 mm wide. Its width remains constant up to the distal end where it flares slightly up to 2.2. A second less complete termination flares to 2.6 mm from a subtending axis 1.0 mm wide. The axes then give the impression of being subdivided into several elongated structures that we interpret as synangiate sporangia.

Several terminal structures are preserved (Fig. 10a–g). The distal end of the ultimate axis is marked by a slight and progressive widening (Fig. 10b–e). The axis then gives rise to several elongate sporangia that are all attached at the same level (Fig. 10d,e). The detailed organization of the termination is difficult to decipher. Although truncated, individual sporangia are particularly visible on the lower specimen in Fig. 10b. In this case three sporangia are visible and separated by a darker line of sediment. In other specimens, up to 4 sporangia can be identified (Fig. 10d,e). Distally, several additional tips can be seen suggesting that there are more sporangia hidden behind. Three to four additional tips can be identified in some cases (Fig. 10d,e). We consequently interpret the termination as being of 6 to 8 sporangia very likely organized in a circle. Individual sporangia are 0.4 to 0.8 mm wide and 4–6 mm long. They are spindle-shaped in profile, truncated proximally at the site of attachment and tapering distally to an acute tip. In some cases a slightly darker line can be observed within the sporangia. It could be interpreted as a longitudinal dehiscence line (Fig. 10c).

*Yarravia*^64^

*Yarravia oblonga*^64^

Fig. 10h and i

### Material

One specimen from the LPL.

### Description

This plant consists of robust smooth parallel-sided axes (Fig. 10h). Only one dichotomy is preserved. The axes are terminated by a truss of sporangia resembling a synangium. This specimen is 23 mm long and only shows the ultimate dichotomy and the axes subtending the terminal structures. The penultimate axis order is 9.8 mm long and 0.8 mm wide. It dichotomizes at an angle of 58° forming two axes measuring 4.5 and 7.0 mm long and 1.0 and 1.1 mm wide respectively. Only one synangium-like structure is preserved. Just before the insertion point of the sporangia, the subtending axis widens to 2.1 mm. Three elongate sporangia appear to all be inserted at the same level and are 3.5–4.2 mm long and 0.7–1.0 mm wide (Fig. 10i). They are parallel-sided. Their apices narrow abruptly and terminate in a slightly recurved beak-like structure. The tip of a possible fourth distal sporangium is also apparent.

### Identity and comparison

Plants presenting dichotomizing axes terminated by synangiate structures are rare in the Lower Devonian. The Impofu Dam material strongly recalls material attributed to the genus *Yarravia* that was reported from several Lower Devonian localities in Australia^65,28,57^ . Two species were identified *Yarravia oblonga* and *Yarravia subsphaerica*. The Impofu Dam material conforms more strongly to *Yarravia oblonga.* Of this plant, only the synangium-like structures and part of their subtending axes are known. The subtending axes present a massive aspect like that observed in the Impofu Dam material and the synangium like structures share the same organization. The size of the South African specimen is however smaller than the original material from Yarra Track but conforms almost exactly to material referred to as *Yarravia* cf. *oblonga* from Lilydale. Other occurrences of the genus *Yarravia* have been reported from France and Russia^66,67^, however these specimens need further study in order to be properly compared. Finally, specimens attributed to the genus *Yarravia* have been collected from the Devonian of Arizona but would as well need additional investigation^68^.

Incertae sedis heart-shape termination.

Fig. 10j and k.

### Material

A single specimen of this plant has been recovered from LPL.

### Description

The plant measures 47 mm long. The branching system is apparently isotomous and branches only once (Fig. 10j). The first order is 33 mm long and 0.6 mm wide, slender and smooth. Second order are 9.2 and 9.8 mm long and 0.5 mm wide. The apex of each ultimate axis consists of a heart-shape structure measuring 4.0 mm long and 2.3 to 2.6 mm in width (Fig. 10k). This structure is complex and comprised of at least two more or less independent units. The two structures are easy to distinguish however they never seem to separate completely before their tips. These structures, here interpreted as sporangia, seem to occur after a dichotomy of the axis. Each one then progressively widens to reach 1.0 to 1.3 mm wide at two third of its length. Thereafter it forms a rounded tip, giving to the whole sporangium a club-shape.

### Identity and comparisons

This plant bears a superficial resemblance to the Australian *Yarravia* Lang and Cookson^28^. *Yarravia* is characterised by several elongated sporangia apparently all attached at a single point. Our material rather seems to be composed of only two sporangia. A similar organisation was also described from the Lochkovian north Brown Clee Hill locality, Welsh Borderland (UK)^60–62^ . Although much smaller and preserved anatomically, *Grisellatheca salopensis* Edwards et al.^62^ presents a heart-shape fertile region made of two sporangia. Further comparison is however made difficult by the lack of anatomical details in our material.

## Discussion

### Diversity of the new Baviaanskloof Formation flora

The flora of the Lower Devonian Baviaanskloof Formation is remarkably diverse. The uppermost and the lowermost plant-bearing lenses (UPL and LPL) from Impofu Dam that were investigated tend to show a relatively high taxonomic diversity. The two plant-bearing lenses show slightly different assemblages. Smaller plants are present in the LPL which are often less fragmentary and generally better preserved. The UPL includes a greater proportion of larger plants which are frequently more fragmentary. Transport could therefore be responsible for some selection and sorting of material. The time span between the moment of deposition of the two plant levels is impossible to assess reliably but it may also have affected the differences in diversity between the two plant levels. In addition, slightly different environments may be recorded with resultant palaeoecological variation in plant assemblages. Wellman et al.^42^ provided evidence for such variations in the Anglo-Welsh basin. They suggested that plant distribution in the earliest continental ecosystems was affected by the substrate composition and by the position on the floodplains.

In this regard, it is pertinent to note that the only previously described plant fossils from the Baviaanskloof Formation (namely those described by Höeg in 1930 from the Kareedouw Member outcrop in the Blaaukrantz River pass near Plettenberg Bay, South Africa) are attributed to *Dutoitia pulchra*, which is absent from the two assemblages at Impofu Dam.

### Characteristics of the Baviaanskloof flora

Up to now, 15 taxa have been identified during this work. A large quantity of additional material is still under study and includes several yet unresolved specimens and gametophytes. *Cooksonia* is by far the most diverse element of the here reported flora with at least three species having been identified. Despite recent revision some questions remain regarding its taxonomy. Gonez and Gerrienne^6^ proposed a new generic diagnosis focusing on sporangial shape. Utilising *C. pertoni* as an example, this diagnosis proposed that only cup- or trumpet-shaped sporangia should be included within *Cooksonia*. This would de facto exclude the poorly known *C. hemisphaerica* and *C. cambrensis* that instead have rounded sporangia. This highlights the challenges existing within early land plant taxonomy. Indeed, vegetative features being uninformative, all taxonomic distinctions are based on, sometimes minute, morphological differences in sporangial architectures. Cryptic taxonomic diversity was notably highlighted by Fanning et al.^69^ who demonstrated that similarly looking *Cooksonia* sporangia can be found to produce different spore types. The preservation of the material in SA unfortunately precludes such assessments but, considering the size range in both sporangia and axes, cryptic diversity may occur as well within the Impofu *Cooksonia* material.

Other rhyniophytoids are also present but in lesser proportions. These include *Uskiella* and *Tortilicaulis* together with a range of other putative taxa exhibiting varying sporangial morphologies.

Regardless of taxonomic uncertainties, several observations are possible.

1. All branched plant remains are characterized by strict isotomous branching.
2. All axes observed in the Impofu Dam flora are smooth.
3. All sporangia are terminal.

All plant remains (except *Sporogonites*) appear to be of a similar grade of organisation, typical of early rhyniophytoids, showing characteristics considered plesiomorphic among embryophyte evolution^23^. Notable exceptions are *Yarravia oblonga* and *Mtshaelo kougaensis* that both show a relatively more complex organisation with the presence of synangiate structures. The branching systems of both plants however remain perfectly isotomous.

### Stratigraphic bearings

The age of the Baviaanskloof Formation has been a matter of uncertainty. The most accepted biostratigraphic constraint has been presence of the Agulhas endemic mutationellid brachiopod, *Pleurothyrella*^13^, commonly associated with the upper unit. Additional invertebrate evidence has seemed to support this age, though similarly all marine invertebrates are only known from the ‘Upper Unit’. This has, nonetheless, been used to suggest a Pragian to Emsian age for the entire Formation.

The here reported flora however challenges these assumptions. Indeed, as already stated, this flora is nearly solely composed of plants at a rhyniophytoid grade of organisation, very characteristic of Silurian/Early Devonian (Lochkovian) floras. *Cooksonia,* that constitutes a major component of the Baviaanskloof flora is a very widespread genus. It has up to now only been found in deposits ranging from the Silurian to the Lochkovian but never in younger horizons. Finally, a more indirect evidence of the age is provided by the lack of zosterophylls and lycophytes in the assemblage. Indeed, paleogeographical reconstructions tend to show that, during the Silurian, zosterophylls and Lycophytes were confined to equatorial environments and during the Lochkovian progressively colonized higher latitudes^8,39^. They reached the Anglo-Welsh basin during mid-Lochkovian times^72^, but are also lacking in the Lochkovian Paraná basin assemblages from Brazil.

Invertebrate fossils observed in the upper unit at Mpofu Dam are consistent with a possible Pragian to Emsian age (pers. comm. Dr Norton Hiller), however our research suggests that this age estimate should not be extended to include the lower portion of the formation, including the Kareedouw Member. We consider that the palaeobotanical information presented here shows several lines of evidence, particularly the similitudes with the Paraná basin assemblage from Brazil (see below), that constrain the age of the middle part of the formation (at least) to the Lochkovian. This assessment is strengthened by inclusion of taxa that are, unlike the invertebrates, not endemic to the Agulhas Basin. It seems likely therefore that the lower portion of the formation is Lochkovian in age, with the ‘upper unit’ representing Pragian deposits. This would be consistent with association of the sudden basinal deepening represented by initiation of Bokkeveld sedimentation, with the Emsian transgression. It would however imply that more gradual basinal deepening preceded this transgression. Ongoing investigation of the biota of the Baviaanskloof Formation are hoped to provide finer scale resolution.

### Comparison with other coeval floras

Land plant megafossils are rarely found among Silurian and Lower Devonian deposits^50,51,63^. To date, only 23 localities are recorded. In most cases, reports consist of monospecific or very poorly diversified assemblages, making broad scale comparison difficult.

The Impofu flora most closely resembles two palaeogeographically very distinct floras. These are the Anglo-Welsh borderland localities assemblage and the Paraná basin assemblage. The Anglo-Welsh basin assemblage is one of the best documented from the Silurian to Lower Devonian time interval^1,59^. Morris and Edwards^72^ highlighted that throughout the Early Devonian, there are changes in plant composition that very likely reflect evolutionary innovations. The Impofu Dam assemblage is best compared with the lower Lochkovian assemblages. The Impofu Dam assemblage is however most similar to the Lochkovian Paraná Basin flora from Brazil^2–6,34,35^.

In the Lower Devonian, Brazil and South Africa were located around the same sedimentary basin^40^, the Agulhas Sea, and very likely shared similar climatic conditions. This paleogeographic and climatic proximity most likely explains the numerous resemblances existing between the two assemblages. By contrast with the Brazilian floras, the Impofu Dam material presents at least one species in common with the Lower Devonian of Australia^28^. Further biogeographic comparisons and discussion is however deferred until description of enigmatic specimens and probable gametophyte material has been completed.

## Conclusions

This paper represents a first account of the newly discovered early land plant bearing lenses from the Baviaanskloof Formation strata at Impofu Dam in the Eastern Cape province of South Africa. It provides evidence for one of the most diverse Late Silurian/Early Devonian assemblages known. Approximately 15 taxa are represented, 14 of which may be reasonably diagnosed. The assemblage bears the greatest resemblance to Early Lochkovian assemblages from the Paraná Basin of Brasil and the Anglo Welsh basin. This study provides reliable biostratigraphic constraints on the dating of the Baviaanskloof Formation, the age of which has been the subject of some debate. Finally, the Impofu Dam assemblage represents the oldest known megaflora from sub-Saharan Africa.

## Data Availability

The material is housed in the collections of the Albany Museum, Somerset Street, Grahamstown, Makhanda, Eastern Cape, South Africa.
